# Latitudinal variation in soil nematode communities under climate warming‐related range‐expanding and native plants

**DOI:** 10.1111/gcb.14657

**Published:** 2019-05-20

**Authors:** Rutger A. Wilschut, Stefan Geisen, Henk Martens, Olga Kostenko, Mattias de Hollander, Freddy C. ten Hooven, Carolin Weser, L. Basten Snoek, Janneke Bloem, Danka Caković, Tatjana Čelik, Kadri Koorem, Nikos Krigas, Marta Manrubia, Kelly S. Ramirez, Maria A. Tsiafouli, Branko Vreš, Wim H. van der Putten

**Affiliations:** ^1^ Netherlands Institute of Ecology Wageningen The Netherlands; ^2^ Laboratory of Nematology Wageningen University Wageningen The Netherlands; ^3^ Theoretical Biology and Bioinformatics Utrecht University Utrecht The Netherlands; ^4^ Department of Biology, Faculty of Natural Sciences and Mathematics University of Montenegro Podgorica Montenegro; ^5^ Biološki inštitut Jovana Hadžija ZRC SAZU Ljubljana Slovenia; ^6^ Department of Botany, Institute of Ecology and Earth Sciences University of Tartu Tartu Estonia; ^7^ Department of Ecology, School of Biology Aristotle University Thessaloniki Greece; ^8^ Institute of Plant Breeding and Genetic Resources Hellenic Agricultural Organization Demeter Thessaloniki Greece

**Keywords:** *Centaurea stoebe*, enemy release hypothesis, plant‐pathogenic nematodes, range expansion, range‐expanding plant species, root‐feeding nematodes

## Abstract

Current climate change has led to latitudinal and altitudinal range expansions of numerous species. During such range expansions, plant species are expected to experience changes in interactions with other organisms, especially with belowground biota that have a limited dispersal capacity. Nematodes form a key component of the belowground food web as they include bacterivores, fungivores, omnivores and root herbivores. However, their community composition under climate change‐driven intracontinental range‐expanding plants has been studied almost exclusively under controlled conditions, whereas little is known about actual patterns in the field. Here, we use novel molecular sequencing techniques combined with morphological quantification in order to examine nematode communities in the rhizospheres of four range‐expanding and four congeneric native species along a 2,000 km latitudinal transect from South‐Eastern to North‐Western Europe. We tested the hypotheses that latitudinal shifts in nematode community composition are stronger in range‐expanding plant species than in congeneric natives and that in their new range, range‐expanding plant species accumulate fewest root‐feeding nematodes. Our results show latitudinal variation in nematode community composition of both range expanders and native plant species, while operational taxonomic unit richness remained the same across ranges. Therefore, range‐expanding plant species face different nematode communities at higher latitudes, but this is also the case for widespread native plant species. Only one of the four range‐expanding plant species showed a stronger shift in nematode community composition than its congeneric native and accumulated fewer root‐feeding nematodes in its new range. We conclude that variation in nematode community composition with increasing latitude occurs for both range‐expanding and native plant species and that some range‐expanding plant species may become released from root‐feeding nematodes in the new range.

## 1 INTRODUCTION

As a consequence of anthropogenic climate change, many species are naturally expanding their native range to higher latitudes or altitudes (Parmesan & Yohe, 2003; Rumpf et al., 2018; Steinbauer et al., 2018). However, range expansion rates may not be uniform across organismal groups because of different dispersal capacities. While microbial organisms below a size of 20 μm may have a very high dispersal capability (Foissner & Hawksworth, 2009; Wilkinson, Koumoutsaris, Mitchell, & Bey, 2012), larger belowground organisms such as nematodes may be more limited in dispersal than aboveground organisms such as plants (Berg et al., 2010). Among these rhizosphere organisms, plant mutualists and antagonists can affect plant performance and vegetation dynamics by growth promotion or reduction (Kardol, Bezemer, & van der Putten, 2006; van der Heijden, Bardgett, & van Straalen, 2008). The disruptions of interactions between plants and such rhizosphere organisms that are caused by varying range expansion rates may therefore have functional consequences for plant performance in the new range (Morriën, Engelkes, Macel, Meisner, & van der Putten, 2010; van der Putten, 2012). Such changes in plant performance between the new and the original range have been observed in the case of plant introductions into novel continents by humans and are attributed to the release from specialized belowground natural enemies that are present in the original range. Indeed, several intracontinental range‐expanding plant species also seem to be less negatively affected by soil communities in their new than in their original range, suggesting that range expansion causes the release from natural enemies of the original range (De Frenne et al., 2014; Dostálek, Münzbergová, Kladivová, & Macel, 2015; van Grunsven, van der Putten, Bezemer, Berendse, & Veenendaal, 2010; Van Nuland, Bailey, & Schweitzer, 2017). However, the actual shifts in soil biota potentially underlying these changes in range‐expanding plant performance have so far not been studied in the field.

Among soil biota, nematodes are the most abundant animals and perform key functions as bacterivores, fungivores, omnivores, predators and root herbivores (de Ruiter, Neutel, & Moore, 1995; Ferris, Bongers, & De Goede, 2001; Yeates, Bongers, Degoede, Freckman, & Georgieva, 1993), but their potential role in the success of range‐expanding plant species is not yet fully understood (Morriën, Duyts, & van der Putten, 2012; Wilschut, Kostenko, Koorem, & van der Putten, 2018). Moreover, while root‐feeding nematodes are well studied with respect to their role as agricultural pests (Nicol et al., 2011), their role in natural ecosystems has received less attention (De Deyn et al., 2003). It has been proposed that changes in nematode community composition, especially lowered exposure to root‐feeding nematodes, may drive the high performance of range‐expanding plant species in new range soils (Engelkes et al., 2008; Morriën et al., 2012). However, as surveys along natural range expansion gradients are lacking, this proposed release of range‐expanding plant species from belowground nematode enemies has not yet been verified.

Survey‐based sampling along latitudinal or altitudinal transects can be useful to help explore potential shifts in nematode community composition, as has been shown for other soil organisms, such as bacteria, fungi and protists (Bahram et al., 2018; Bates et al., 2013; Delgado‐Baquerizo et al., 2018; Tedersoo et al., 2014; Thompson et al., 2017). For nematodes, surveys have been rarely performed due to methodological constraints, as they are highly time‐consuming due to expert‐dependent morphological identification. The few survey‐based studies that have relatively limited sample sizes show that climate, vegetation and soil abiotic conditions co‐determine nematode community composition (Chen et al., 2015; Nielsen et al., 2014; Sylvain et al., 2014), which was supported by a recent meta‐analysis (Song et al., 2017). Both these nematode‐centred surveys, as well as comparable studies on microbial communities, focus on community variation between soils, without considering the influence of plant species on soil communities. Rhizosphere communities, however, can considerably differ between individual plant species (Bezemer et al., 2010). So far, latitudinal patterns in rhizosphere community composition of specific plant species have rarely been studied (Lu, He, Ding, & Siemann, 2018) and not yet in the context of plant range expansions.

Recently, novel molecular nematode community analysis approaches have been developed, so that continental‐scale surveys are now becoming feasible (Geisen et al., 2018; Griffiths, de Groot, Laros, Stone, & Geisen, 2018). We used such a molecular sequencing technique, combined with morphological quantification, in order to perform a high‐resolution analysis of 357 rhizosphere nematode communities along a 2,000 km long latitudinal plant range expansion gradient across Europe. In six countries, rhizosphere samples were collected from four range‐expanding species' original ranges in South‐Eastern and Central Europe, and from their new ranges in North‐Western Europe. We also collected samples from four congeneric plant species that are all native along the entire latitudinal transect, in order to control for the latitudinal variation in nematode community composition along this transect. In addition to assessing the composition of nematode communities, we examined their total abundances, as well as the abundances of nematode feeding types and several root‐feeding nematode groups, which are known to differ in feeding modes and effects on plant performance (Bongers, 1988; Yeates et al., 1993). We explored biogeographical patterns in nematode richness and community composition along the latitudinal transect and tested the hypotheses that (1) latitudinal shifts in nematode community composition are stronger in range‐expanding plant species than in congeneric natives and (2) in their new range, range‐expanding plant species accumulate fewer root‐feeding nematodes than in their original range.

## 2 MATERIALS AND METHODS

### 2.1 Plant species

The range expanders in our study were *Centaurea stoebe* and *Tragopogon dubius* (Asteraceae), *Geranium pyrenaicum* (Geraniaceae) and *Rorippa austriaca* (Brassicaceae). Congeneric native plant species were *Centaurea jacea*, *Tragopogon pratensis*, *Geranium molle* and *Rorippa sylvestris*, respectively. The range expanders naturally occur in southern and/or Central Europe (native range) and have recently new into North‐Western Europe (NDFF, 2017). *C. stoebe*, *T. dubius* and *R. austriaca* colonized North‐Western Europe in the course of the 20th century, while *G. pyrenaicum* was present in the 19th century, but strongly increased in the last decades of the 20th century (NDFF, 2017). The congeneric native plant species naturally occur throughout the entire geographical area examined in this study (NDFF, 2017), and all native plant species were already considered as native to the Netherlands in the early 19th century (Van der Meijden, 2005). In North‐Western Europe, all plant species occur in the same riverine ecosystems, although their specific habitat requirements may differ.

### 2.2 Field sampling

**Figure 1:**
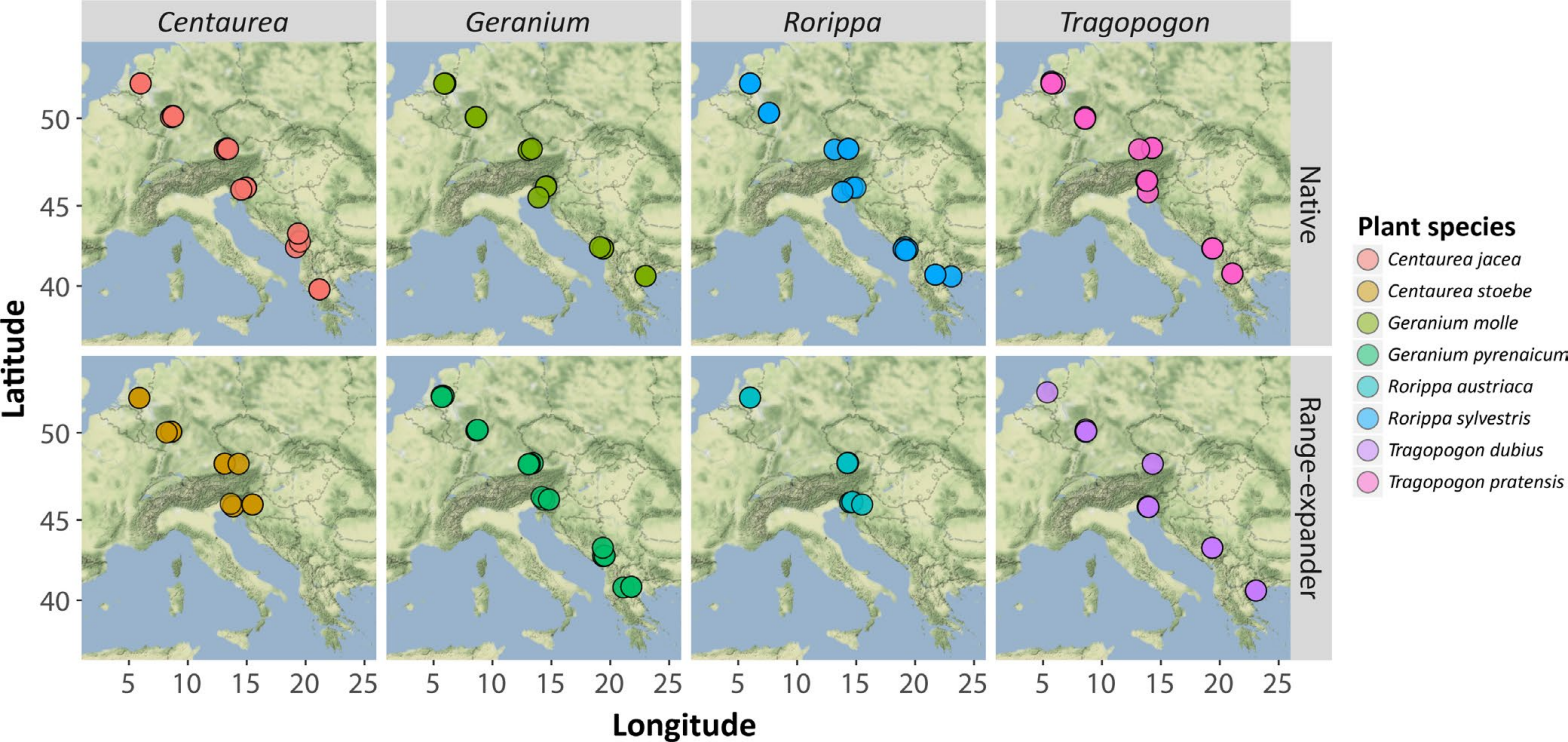
Latitudinal sampling scheme of four native and four range‐expanding plant species in six European countries. For each plant species, coloured circles represent populations within each of the six countries where plants were sampled: Greece (south‐east), Montenegro, Slovenia, Austria, Germany and the Netherlands (north‐west), respectively

In the growing seasons of 2013 and 2014, we collected soil around the roots of flowering individuals of all eight plant species along a latitudinal transect from South‐Eastern to North‐Western Europe, including Greece, Montenegro, Slovenia, Austria, Germany and the Netherlands. In each country, we aimed to sample nine individual plants for each plant species (three individuals per population, with three populations per country) (Figure 1, Figure S1). Populations were separated at least 2 km from each other. One soil sample was collected under each plant individual. As *C. stoebe* and *R. austriaca* do not naturally occur in Greece and Montenegro, these species were only sampled from Slovenia northwards. After collection, soils were stored in transportable coolers and, as soon as logistically possible, at 4°C until nematode extraction. Soil moisture was determined for both the 2013 and 2014 samples. Additionally, for all 2014 samples, we measured pH, C/N ratio and the content of plant‐available ${\text{NH}}_{4}^{+}$, ${\text{NO}}_{2}^{-}+{\text{NO}}_{3}^{-}$ and phosphate (see below).

### 2.3 Soil abiotics

To not limit our understanding of nematode community composition to the effects of plant species and latitude, we also measured important soil characteristics. To determine soil moisture (water % w/w), fresh soil samples were dried at 105°C for 12 hr. Subsequently, this dried soil was ground using a ball mill, and 5 mg was weighted into tin cups in order to determine total soil C and N content using an elemental analyser (LECO). For other measurements, soil samples were dried at 40°C for 5 days. Plant‐available phosphate (P‐Olsen) was measured by extraction from 2.5 g of soil in a 0.5 M NaHCO_3_ solution and quantification using an auto‐analyser (QuAAtro Autoanalyzer; SEAL Analytical Ltd.). Available mineral nitrogen (${\text{NH}}_{4}^{+}$ and ${\text{NO}}_{2}^{-}+{\text{NO}}_{3}^{-}$) was measured using the KCl extraction protocol: dried soil samples of 10 g were shaken in a 1 M KCl solution for 2 hr, after which concentrations were determined using an auto‐analyser (QuAAtro Autoanalyzer; SEAL Analytical Ltd.). Soil pH was determined from the KCl extracts.

### 2.4 Nematode extraction and quantification

Stones and other large particles were removed from the collected soil samples, after which approximately 100 g of soil was used for extraction. Nematodes were extracted from a weighed amount of soil using Oostenbrink elutriators (Oostenbrink, 1960). Suspensions (10 ml) with extracted nematodes were divided into two subsamples: one subsample was used for DNA extraction and amplicon sequencing (see below), while the other was used for nematode quantification by visual counting. Before nematode counting, these suspensions were concentrated to 2 ml, after which 4 ml hot (90°C) and 4 ml cold (20°C) formaldehyde were added to heat‐kill and fixate the nematodes. Total numbers of nematodes were then counted using an inverse light microscope (200×; Olympus CK40), and nematode numbers were expressed per 100 g of dry soil using the soil moisture data.

### 2.5 DNA extraction and amplicon sequencing

DNA from the subsample was extracted using the Clear Detections Nematode DNA extraction and purification kit™ (Clear Detections). DNA isolates were stored at −20°C until further use. To obtain taxonomic information on the complete soil nematode community, we amplified the most variable part of the 18S rDNA, the V4 region (Pawlowski et al., 2012) using the universal eukaryotic primers 3NDf together with 1132rmod as previously described (Geisen et al., 2018). We used pretagged primers with Illumina adapters, a 12 bp long barcode to allow de‐multiplexing of the reads after sequencing, a primer linker and the sequencing primers. All PCRs were conducted in duplicate, product quality was visually verified on agarose gel, and duplicates were pooled before PCR clean‐up with Agencourt AMPure XP magnetic beads (Beckman Coulter). PCR cycling conditions were as follows: initiation for 5 min at 94°C, followed by 35 cycles of 45 s at 94°C, 1 min at 53°C and 90 s at 72°C with a final elongation for 10 min at 72°C. PCR products were pooled in equimolar ratios after determining concentrations with a fragment analyser (Advanced Analytical) and sent for sequencing to BGI, China.

### 2.6 Bioinformatics

The obtained raw 18S rDNA sequence reads were curated in the Hydra pipeline (de Hollander, 2017) implemented in Snakemake (Köster & Rahmann, 2012); in short, after filtering contaminants and removing barcodes, the forward reads were used for annotation. Thereafter, vsearch (Rognes, Flouri, Nichols, Quince, & Mahé, 2016) was used to cluster all reads into operational taxonomic units (OTUs) using the UPARSE strategy by de‐replication followed by sequence‐sorting by abundance (singletons were removed) and clustering using the UCLUST smallmem algorithm (Edgar, 2010). Chimeric sequences were removed using UCHIME (Edgar, Haas, Clemente, Quince, & Knight, 2011), as implemented in vsearch. To create an OTU table, all reads were mapped to OTUs using the usearch_global method (vsearch). Sequences were aligned to the PR2 database (Guillou et al., 2013). Reference sequences were first trimmed with forward and reverse primers using cutadapt (Martin, 2011). Moreover, we deleted all reference sequences of environmental nematode DNA, to improve annotation success. Prior to further analyses, we removed samples with fewer than 1,000 reads. We then recalculated read numbers to relative abundances of the OTUs. OTUs that could be assigned to nematode genera allowed estimates of relative abundances of functional groups (Yeates et al., 1993). Sequence data were uploaded to the European Nucleotide archive under entry number PRJEB32145.

### 2.7 Statistical analyses

For both multivariate and univariate analyses, nematode communities collected under individual plants were treated as independent samples. Distances between collected plants were highly variable among populations. Therefore, population was not included as a factor in our models.

### 2.8 Multivariate analyses of nematode community composition

Prior to multivariate analyses, we assembled two databases. One database consisted of the relative abundance data of all sequenced nematode OTUs, whereas the other contained relative abundance data of nematode genera based on the sequenced OTUs that could be assigned to nematode genus level. This genus‐level database was assembled to obtain a better indication of functional dissimilarity of the nematode community, as nematode species from the same genus tend to have similar ecological functions, especially in feeding behaviour (Yeates et al., 1993). All multivariate analyses were performed in canoco 5 (Šmilauer & Lepš, 2014; Ter Braak & Šmilauer, 2012), and all analyses were performed for both the OTU‐level and genus‐level data sets.

With all samples collected in 2014, for which soil characteristics were measured, we first ran forward selection RDAs to estimate the importance of the nominal factor (plant species) and the continuous variables (latitude, soil moisture, pH, soil C/N ratio, ${\text{NH}}_{4}^{+}$, ${\text{NO}}_{2}^{-}+{\text{NO}}_{3}^{-}$ and available phosphate) to the variation in nematode community composition. All factors or variables explaining at least 5% of the total variation explained by the RDA model were included in principal component analyses (PCAs) to visualize their contribution to the separation of the samples. Subsequently, using the combined 2013 and 2014 data, we tested for each plant pair (i.e. *C. stoebe* and *C. jacea*) whether range‐expanding plant species showed more profound differences in nematode community composition between the different parts of the geographical range compared to the congeneric native plant species. For this, we combined the country data to compose three regions based on latitude: south (Greece and Montenegro), central (Slovenia and Austria) and north (Germany and the Netherlands). To examine the differences in nematode community shifts, we visualized variation in the community composition using PCAs per plant pair and tested the plant*region interaction using RDAs.

### 2.9 Univariate analyses of functional group abundance

Using the combined 2013 and 2014 data, we performed separate analyses of nematode abundances by comparing range‐expanding and congeneric native plant species for each plant genus, as not all plant species were collected in each latitudinal region. Prior to univariate analyses, we assigned each nematode genus detected by the 18S sequencing to one of the functional groups described in Yeates et al. (1993): bacterivores, fungivores, root‐feeders or the combined group of omnivorous and predatory nematodes. Additionally, the group of root‐feeding nematodes was divided into endoparasites, semi‐endoparasites, ectoparasites and root‐hair feeders. To obtain absolute abundance data, relative abundances of all groups were corrected using the total nematode counts for each sample. Per plant pair, we then modelled absolute abundances (per 100 g dry soil); relative abundances (fraction of total nematode 18S rDNA reads) of bacterivorous, fungivorous, predatory–omnivorous and root‐feeding nematodes; and absolute abundances of the four groups of root‐feeding nematodes. Absolute abundances were treated as count data and converted to integer values, as required in count data analyses. All subsequent analyses were performed in R (R Core Development Team, 2012). To account for overdispersion, abundance data were modelled using generalized linear models with a negative binomial distribution, *glm.nb* in mass (Ripley et al., 2013), which included species, region and the species*region interaction as fixed factors. Post hoc Wald tests were performed using the phia package (De Rosario‐Martinez, 2013). Models were validated by inspection of residual plots. Relative abundance data were modelled with general linear models (*lm* in the stats package), including the same factors as the models for absolute abundance data.

## 3 RESULTS

Visual quantification showed that on average, soil samples contained 2,924 (±151) nematodes per 100 g dry soil. Nematode abundances were not correlated with latitude (Figure S1A). Total nematode abundances varied between plant species, with the highest number found in the range expander *G. pyrenaicum* and the lowest in the range expander *C. stoebe* (Figure S1B).

**Figure 2:**
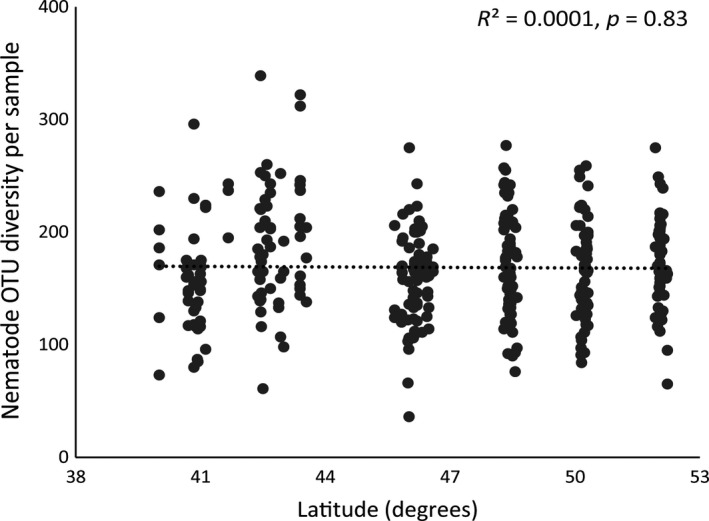
Overall correlation between latitude (degrees) and nematode operational taxonomic unit (OTU) richness per sample along a latitudinal transect from Greece to the Netherlands. Samples represent four range‐expanding plant species and four congeneric related natives (also see Figure S2). *R* ^2^ and *p*‐value of the Pearson correlation test are shown

Overall, 5,368,503 sequences (average of approximately 15,000 sequences per sample) were obtained after removing samples that contained less than 1,000 reads. A total of 961 OTUs were detected, with 653 being assigned as nematodes. More than half of these OTUs could be classified reliably into 92 nematode genera, while 297 OTUs were not assignable to a genus. On average, samples contained 169 (±2.4) OTUs. Overall, OTU richness did not vary with latitude (Figure 2), and only in *T. pratensis,* a weak positive correlation between latitude and nematode OTU richness was found (Figure S2).

### 3.1 Drivers of nematode community composition

#### 3.1.1 OTU‐level community composition

**Figure 3:**
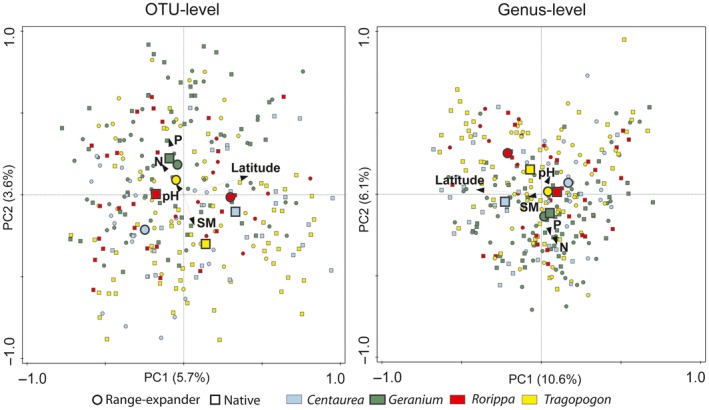
Ordination plots based on principal component analyses (PCAs) of nematode communities, on operational taxonomic unit (OTU)‐level (left) or genus‐level (right). Black arrows represent the effects of continuous variables such as latitude, soil moisture (SM), pH, the available NO_3_ + NO_2 _(N) and phosphorus (P). Large circles and squares represent centroid plant species effects of range‐expanding *Centaurea stoebe*, *Geranium pyrenaicum*, *Rorippa austriaca* and *Tragopogon dubius* and native plant species *Centaurea jacea*, *Geranium molle*, *Rorippa sylvestris* and *Tragopogon pratensis*, respectively. Individual samples of each plant species are indicated with small circles (range expanders) or squares (natives)

All factors and variables included in the RDAs together explained 13.3% of the total variation in the nematode community composition (overall model: pseudo‐*F* = 3.7, *df* = 14, *p* < 0.01). Nematode community composition was significantly affected by plant species, and the majority of the plant species contributed at least 5% to the variation explained by the fitted factors in the RDA model (Table S1), with all included plant species together explaining 5.5% of the total variation in community composition. In particular, the nematode community composition under *C. stoebe* was significantly distinct from other plant species (explained variation: 1.2%; *F* = 3.5; *p* < 0.01; Figure 3; Table S1). Nematode community composition also significantly changed with latitude, which was the strongest continuous explanatory variable (explained variation: 2.1%, *F* = 6.0; *p* < 0.01; Figure 3), followed by available phosphate (explained variation: 1.3%; *F* = 3.7; *p* < 0.01; Figure 3; Table S1). Latitude corresponded with the first PCA axis, whereas available phosphate and soil moisture corresponded most strongly with the second PCA axis (Table S1, Figure 3).

#### 3.1.2 Genus‐level community composition

All factors and variables included in the RDAs together explained 15.1% of the variation in nematode community composition (overall model pseudo‐*F* = 2.9, *df* = 14, *p* < 0.01). Similar to the OTU‐level community composition, latitude (explained 3.1% of the total variation, *F* = 8.9; *p* = <0.01, Figure 3) and available phosphate (explained 1.6% of the total variation, *F* = 4.7; *p* = <0.01, Figure 3) were the continuous variables that explained most of the variation in nematode community composition (Table S1). In contrast to the OTU‐level community composition, single plant species effects were weaker when the community composition was based on nematode genera (Table S1). Latitude also corresponded to the first PC axis and available NO_3_ + NO_2 _(N) to the second PC axis in the ordination of the genus‐level nematode community composition (Table S1, Figure 3).

### 3.2 Nematode community composition shifts between original and new ranges

**Figure 4:**
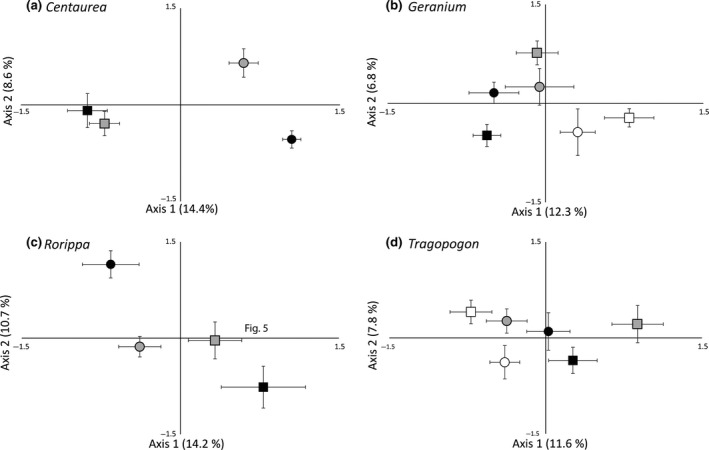
Ordination plots based on principal component analyses (PCAs) of operational taxonomic unit (OTU)‐level nematode communities in the rhizospheres of range‐expanding (circular centroid) and native (rectangular centroid) species of *Centaurea* (a), *Geranium* (b), *Rorippa* (c) and *Tragopogon* (d). Sign colours represent southern latitude soils (white; Greece and Montenegro), central latitude soils (grey; Slovenia and Austria) and northern latitude soils (black; Central‐West Germany and the Netherlands). Error bars represent standard errors of PCA sample scores

For the pair of *Centaurea* species, the differences between OTU‐level nematode communities between the central and northern latitude regions depended on the plant species (Figure 4a; RDA plant species*latitude (spec*lat) interaction: explained variation = 13.3%, *df* = 3, pseudo‐*F* = 3.6, *p* < 0.01). In particular, the nematode community composition under *C. jacea* appeared to differ more strongly between the central and northern regions than under *C. stoebe* (Figure 4a). For the *Geranium* pair, southern OTU‐level nematode communities of native *G*. *molle* differed from nematode communities in the other regions, whereas such differences were not evident for range‐expanding *G. pyrenaicum* (Figure 4b; RDA spec*lat: explained variation = 12.1%, *df* = 5, pseudo‐*F* all axes test = 2.6, *p* < 0.01). In the *Rorippa* species pair, OTU‐level nematode communities differed between the plant species in the northern latitude region, but not in the central latitude region (Figure 4c; RDA spec*lat: explained variation = 14.4%, *df* = 3, pseudo‐*F* all axes test = 2.4, *p* < 0.01). OTU‐level nematode communities of native *Tragopogon* significantly differed between southern and both central and northern latitude regions, while such a separation was not evident in range‐expanding *Tragopogon* (Figure 4d; RDA interaction spec*lat: explained variation = 12.9%, *df* = 5, pseudo‐*F* all axes test = 2.6, *p* < 0.01).

The differences in composition of genus‐level nematode communities between plant species and latitude regions were mostly similar to the differences between OTU‐level nematode communities (Figure S3). Most notably, in *Centaurea,* genus‐level community composition differed significantly between the central and northern latitude regions for range‐expanding *C. stoebe*, while they were comparable in the case of congeneric native *C. jacea* (Figure S3; RDA spec*lat: explained variation = 17.1%, *df* = 3, pseudo‐*F* all axes test = 4.6, *p* < 0.01). Also for the *Rorippa* species, the separation of genus‐level nematode communities between the central and northern latitude regions appeared to be more separated for the range‐expanding *R. austriaca* than for native *R. sylvestris* (Figure S3; RDA spec*lat: explained variation = 14.6%, *df* = 3, pseudo‐*F* all axes test = 2.4, *p* < 0.01).

### 3.3 Abundances of nematode feeding groups

**Figure 5:**
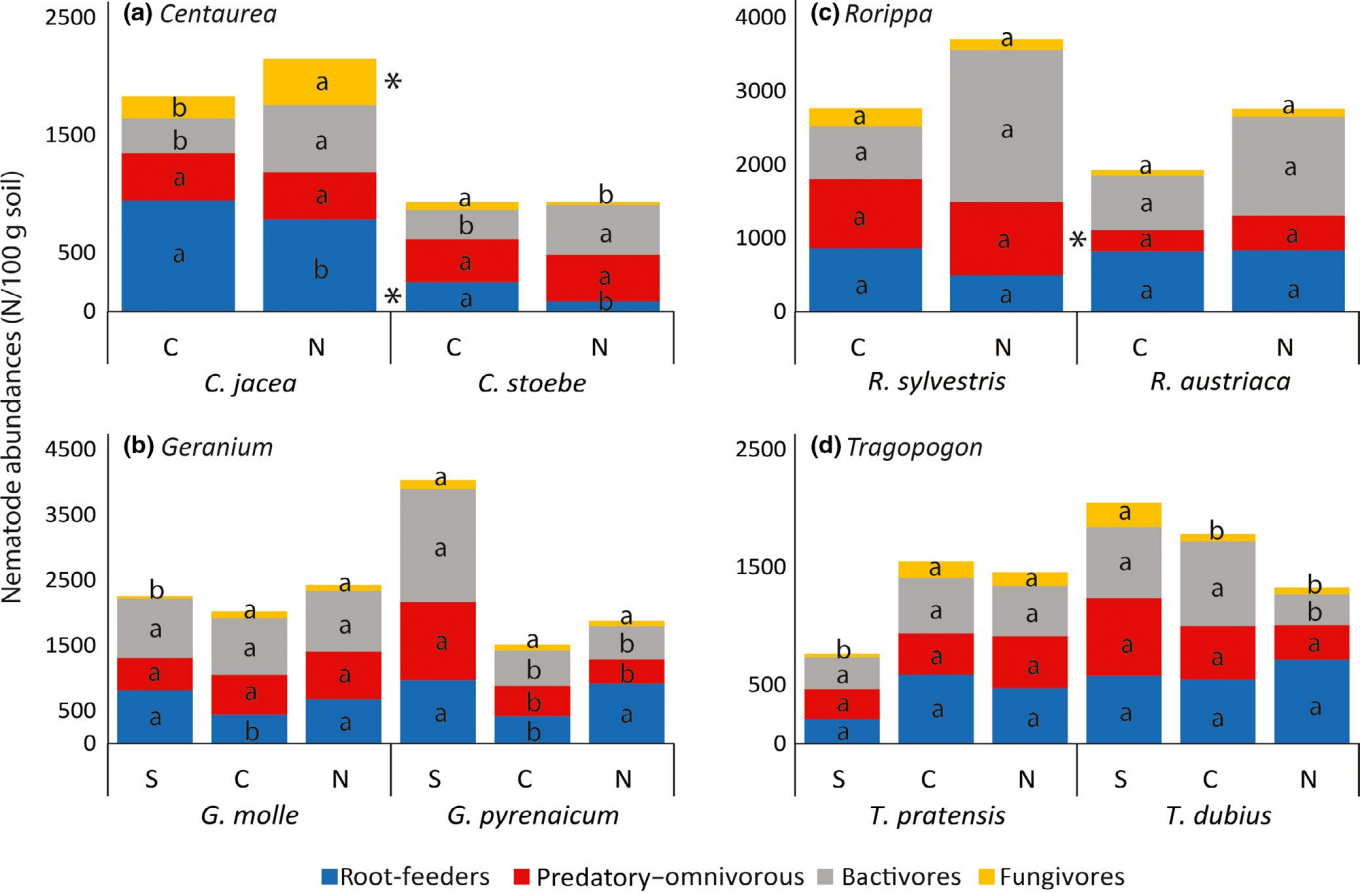
Absolute abundances (number of individuals per 100 g dry soil) of four major nematode feeding groups in rhizosphere samples of range‐expanding *Centaurea stoebe*, *Geranium pyrenaicum*, *Rorippa austriaca* and *Tragopogon dubius* and congeneric native plant species *Centaurea jacea*, *Geranium molle*, *Rorippa sylvestris* and *Tragopogon pratensis* in southern (S: Greece and Montenegro; only *Geranium* and *Tragopogon*) and central (C: Slovenia and Austria; all plant genera) original range soils, and in new range soils (N: Central‐West Germany and the Netherlands; all plant genera). Small letters indicate significant within‐plant‐species differences between regions along the latitudinal gradient according to post hoc Wald tests for each feeding group. Significant between‐plant‐species differences in nematode feeding type abundances are indicated with *

The abundances of nematode feeding groups depended on the plant species and/or latitude region, and none of the feeding groups showed systematic differences between the range‐expanding plant species and the congeneric native (Figure 5). In *Centaurea* (Figure 5a)*,* absolute abundances of root‐feeding nematodes were consistently higher in native *C. jacea* than in range‐expanding *C. stoebe* (*Χ* ^2^ = 34.2, *df* = 1, *p* < 0.001) and consistently lower in the north than in the centre of the latitudinal transect (*Χ* ^2 ^= 4.2, *df* = 1, *p* < 0.05). Moreover, the latitude effect on absolute abundances of fungivores depended on the plant species (*Χ* ^2^ = 10.9,* df* = 1, *p* < 0.001); more specifically, nematode communities of native *C. jacea* had more fungivores in northern than in central latitude regions, while fungivore abundance under *C. stoebe* was lowest in the north (Figure 5a).

In *Geranium*, absolute root‐feeding nematode abundances were lowest at central latitudes, irrespective of plant species (*Χ* ^2 ^= 7.8, *df* = 2, *p* < 0.05; Figure 5b). Rhizospheres of *G. pyrenaicum* contained most predatory–omnivorous and bacterivorous nematodes in the south, whereas this was not the case for *G. molle* (spec*lat effect; predatory–omnivorous nematodes: *Χ* ^2 ^= 17.64, *df* = 2, *p* < 0.001; bacterivorous nematodes: *Χ* ^2 ^= 13.56, *df* = 2, *p* < 0.01; Figure 5b). In *G. molle,* fungivores were least abundant in the south, whereas there was no latitudinal pattern for *G. pyrenaicum* (spec*lat effect: *Χ* ^2 ^= 8.24, *df* = 2, *p* < 0.05; Figure 5b).

For *Rorippa*, the range expander *R. austriaca* had lowest number of predatory–omnivorous nematodes (*Χ* ^2 ^= 10.77, *df = 1,* *p* < 0.01; Figure 5c). The other nematode feeding groups did not show significant differences in absolute abundance between species or latitude regions. In samples of *Tragopogon,* species effects on absolute abundances of fungivores and bacterivores depended on the latitude region: at southern latitudes, fungivores were lowest in *T. pratensis* but highest in *T. dubius* (*Χ* ^2 ^= 18.94, *df* = 2, *p* < 0.001; Figure 5d). Bacterivore numbers were lowest in northern Europe in *T. dubius*, but not in *T. pratensis* (*Χ* ^2^ = 6.30, *df* = 2, *p* < 0.05; Figure 5d).

Analyses of relative abundances of the different nematode feeding types (Figure S4) revealed patterns that were comparable to the analyses of total abundances (Figure 5). Most importantly, although some nematode groups (e.g. predatory–omnivorous nematodes in *Centaurea*) showed significant differences in relative abundance between plant species or latitude regions, these patterns were not necessarily the same when absolute abundances were analysed.

### 3.4 Abundances of root‐feeding nematode types

**Figure 6:**
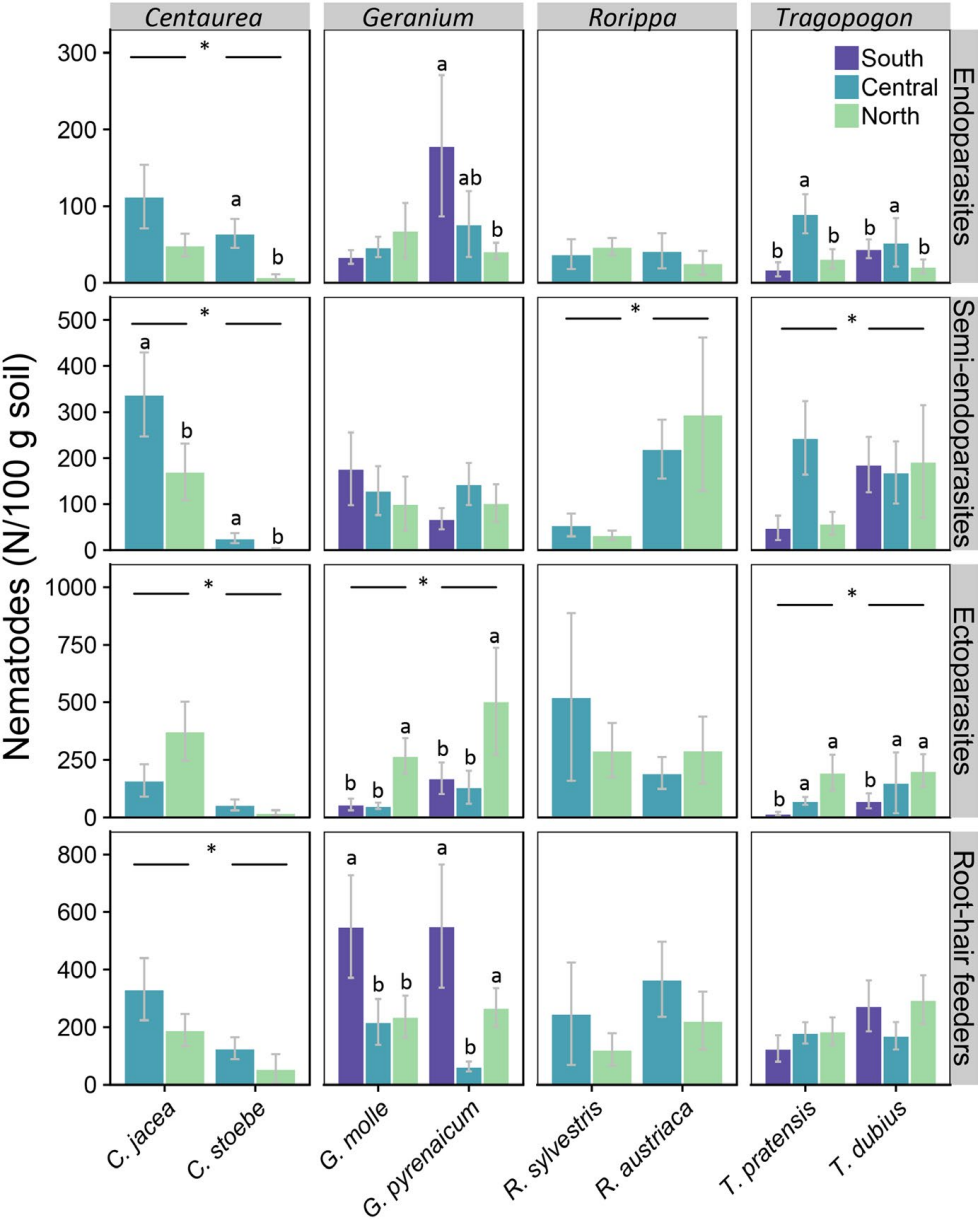
Absolute abundances (number of individuals per 100 g dry soil) of four root‐feeding nematode types in rhizosphere samples of four pairs of congeneric native (left) and range‐expanding (right) species in southern (S: Greece and Montenegro; only *Geranium* and *Tragopogon*) and central (C: Slovenia and Austria; all plant genera) original range soils and in northern range soils (N: Central‐West Germany and the Netherlands; all plant genera). Overall species effects are noted with * and horizontal bars. Significant species*range interactions and range effects are visualized with small letters based on negative binomial GLM and post hoc Wald tests

All four root‐feeding nematode types were more abundant in native *C. jacea* than in range‐expanding *C. stoebe* (single species effects: *Χ* ^2 ^> 9.89, *p*‐values < 0.01), but latitude effects depended on the type of root‐feeding nematode (Figure 6). The abundance of endoparasitic nematodes was lower in the new range (northern latitude region) than in the original range (central latitude region) of *C. stoebe*, while there was no difference between latitude regions in *C. jacea* (spec*lat: *Χ* ^2^ = 3.60, *df* = 1, *p* = 0.058; Figure 6). In both species, the abundance of semi‐endoparasites was lower in the northern than in the central latitude region, but the magnitude of decrease did not significantly differ between species (lat: *Χ* ^2 ^= 9.82, *df* = 1, *p* < 0.01; Figure 6). Moreover, whereas numbers of ectoparasitic nematodes tended to be highest in the new range in *C. jacea* samples, the opposite pattern was observed in *C. stoebe* samples (spec*lat: *Χ* ^2^ = 4.55, *df* = 1, *p* < 0.05; Figure 6).

In samples of range‐expanding *G. pyrenaicum*, there were significantly fewer endoparasites in northern than in southern latitude samples, whereas this was not found for native *G. molle* (spec*lat: *Χ* ^2^ = 9.17, *df* = 2, *p* < 0.05; Figure 6). Abundance of ectoparasites on *Geranium* was highest in the north (lat: *Χ* ^2^ = 20.9, *df* = 2, *p* < 0.0001; Figure 6) and higher in *G. pyrenaicum* than in *G. molle* samples (spec: *Χ* ^2^ = 8.53, *df* = 1, *p* < 0.01; Figure 6). Finally, the latitudinal variation in numbers of root‐hair feeders depended on plant species: in *G. molle,* there were most root‐hair feeders in the south, while in *G. pyrenaicum*, root‐hair feeders were least abundant in centre of the latitudinal transect (spec*lat: *Χ* ^2^ = 6.15, *df* = 2, *p* < 0.05; Figure 6).

In *Rorippa*, semi‐endoparasites were most abundant in *R. austriaca* samples (spec: *Χ* ^2^ = 12.83, *df* = 1, *p* < 0.0001; Figure 6), whereas abundances of other root‐feeding nematodes did not differ between these species. Both *Tragopogon* species had most endoparasites at central latitudes (lat: *Χ* ^2^ = 8.83, *df* = 2, *p* < 0.05; Figure 6). Latitude region also significantly affected the abundance of semi‐endoparasites (lat: *Χ* ^2^ = 6.1, *df* = 2, *p* < 0.05), but the effect was too small to be significant in the post hoc analyses (Figure 6). There were more semi‐endoparasites (spec: *Χ* ^2^ = 4.47, *df* = 1, *p* < 0.05) and ectoparasites (spec: *Χ* ^2^ = 4.2, *df* = 1, *p* < 0.05) in samples of range‐expanding *T. dubius* than in native *T. pratensis* (Figure 6). Both species accumulated most ectoparasites in the north (lat: *Χ* ^2^ = 15.36, *df* = 2, *p* < 0.001; Figure 6).

## 4 DISCUSSION

Our results show soil nematode community composition changes along a latitudinal gradient, but not in nematode richness and abundance. Latitudinal shifts in nematode community composition in general were not stronger for range‐expanding plant species than for the congeneric natives. Only one out of the four range‐expanding plant species, *C. stoebe*, experienced a strong shift in nematode community composition and also accumulated fewer individuals of several root‐feeding nematode groups in its new range.

Our results clearly demonstrate that nematode richness and total nematode numbers did not change with latitude across our sampling range, which is in line with previous studies (Kerfahi et al., 2016; Song et al., 2017). Changes in nematode community composition with latitude may be caused by variation in climatic variables along the latitudinal transect, such as temperature and precipitation (Bhusal, Tsiafouli, & Sgardelis, 2015; Nielsen et al., 2014; Song et al., 2017). Overall, plant species identity was the strongest predictor of nematode community composition in this study. The importance of plant type for nematode community composition has been shown in a previous study (Song et al., 2017), but our study highlights that even between closely related plant nematode communities may be distinct. Opposite to bacteria and fungi (Fierer, Strickland, Liptzin, Bradford, & Cleveland, 2009; Lauber, Hamady, Knight, & Fierer, 2009; Tedersoo et al., 2014), soil characteristics, such as pH and soil moisture, were less important in explaining nematode community composition. Available phosphate appeared to be more important for nematode community composition than other soil characteristics, which corresponds with previously observed effects of fertilizers on nematode community composition (Hu & Qi, 2010; Zhao et al., 2014). Our selection of collection sites that were mainly situated in riverine areas was aimed at minimizing variation in soil conditions. Therefore, we cannot conclude that soil abiotics are generally unimportant for nematode community structuring. Yet, even with this restricted sampling regime, our models explained less than 15% of total variation in nematode community composition, leaving the majority of variation unexplained. Various factors could have contributed to this result. Most importantly, stochastic processes may be an important determinant of nematode community composition at local scales (Quist, 2017), partly because taxa of large‐sized omnivorous and predatory nematodes generally occur in low abundances (Quist et al., 2017). This is further strengthened by strong spatial and temporal dynamics in nematode distribution on local scales (Ettema, Rathbun, & Coleman, 2000). Secondly, while we determined several important soil characteristics, the inclusion of other soil variables, such as soil clay content (Dassen et al., 2017), might have increased the explanatory power of our analyses.

The latitudinal variation in nematode community composition supports our assumption that plant species will face partly different nematode communities during range expansion. It also shows that nematode communities to which widely distributed plant species are exposed vary across their native range. However, as the overall latitudinal variation in nematode community composition was not very strong, it remains unclear whether such shifts in nematode communities could have functional consequences for plant performance. We found mixed support for the hypothesis that shifts in nematode community composition across the latitudinal gradient are stronger for range‐expanding plant species than for congeneric natives (Hypothesis 1). One of the four range‐expanding plant species, *C. stoebe,* showed a stronger shift in genus‐level nematode community composition between the original range in Central Europe and the new range in North‐Western Europe than the congeneric native *C. jacea*. These results suggest different responses of nematodes to range‐expanding *C. stoebe* in the new compared to the original range, as has also been demonstrated for its seed‐ and root‐associated fungal communities (Geisen et al., 2017). For the other three range‐expanding plants, differences in nematode community between the new and the original range were not so strong.

*Centaurea stoebe* was the only range expander that hosted lower numbers of root‐feeding nematodes in the new than in the original range (Hypothesis 2). Specifically, numbers of endoparasites were reduced in the new range, while such a strong decrease was not evident for native *C. jacea*. These low numbers of endoparasitic root‐feeding nematodes in the rhizosphere of *C. stoebe* in the new range might be explained by the strong chemical nematode repellence of *C. stoebe* (Wilschut, Silva, Garbeva, & van der Putten, 2017), which may be stronger in the new range, where root‐feeding nematodes are nonadapted to range‐expanding plant species, than in the original range, where root‐feeding nematodes are adapted to these plant species. Such “novel weapon” effects have previously been shown for the interactions of introduced exotic plant species with aboveground insect herbivores in their new range (Schaffner et al., 2011), but evidence is still lacking in the case of belowground plant–herbivore interactions of non‐native plant species. Overall, however, the reduction of root‐feeding nematodes between the new and the original range was not stronger for *C. stoebe* than for *C. jacea*. This pattern of reduced endoparasitic—but not total—root‐feeding nematode numbers corresponds with a study showing that an invasive exotic grass accumulated low numbers of endoparasites in its novel range (van der Putten, Yeates, Duyts, Reis, & Karssen, 2005), and indicates that not all root‐feeding nematode types will show the same response to plant range expansions. Understanding the functional consequences of this variability will require inoculation experiments under more controlled conditions. The absence of evident changes in root‐feeding nematode numbers in the other three range‐expanding plant species may be due to chemical similarity of their roots to the roots of congeneric native species, such as has been shown for range‐expanding and native *Geranium* and *Rorippa* species (Wilschut et al., 2017). Overall, latitudinal shifts in nematode community composition of range‐expanding plant species do not necessarily imply reduced exposure to root‐feeding nematodes, as this happened in one out of the four range expanders.

While there were no strong latitude effects on numbers of bacterivorous and omnivorous‐predatory nematodes in range‐expanding plant species, numbers of fungivorous nematodes varied along the latitudinal transect: both range‐expanding *Centaurea* and *Tragopogon* species accumulated fewer fungivorous nematodes in northern latitude sites than in central and southern sites, whereas the opposite was found for their congeneric natives. These results correspond with a previous study under controlled greenhouse conditions showing lower abundances of fungivores in the rhizospheres of range expanders than of natives (Morriën et al., 2012). Possibly, this effect can be explained by inhibitory effects of the range expanders on soil fungi in the new range (Morriën & van der Putten, 2013), which has also been shown for introduced non‐native species (Callaway et al., 2008).

We analysed nematode community composition by molecular taxonomic classification often beyond genus‐level using high‐throughput sequencing, in combination with a visual counting of total numbers. An approach with only molecular analyses would have limited comparisons to relative abundances, rather than enabling quantitative analyses (Geisen et al., 2018; Vandeputte et al., 2017). However, we acknowledge that the community composition may not be entirely identical to the result of morphological identification (Darby, Todd, & Herman, 2013; Griffiths et al., 2018). For example, we found relatively high numbers of large‐sized omnivores and predators compared to studies based on morphological identification (e.g. Song et al., 2017), suggesting that HTS might provide community structure information that is more closely representing biomasses rather than abundance information (Zhu, Massana, Not, Marie, & Vaulot, 2005). This could also have led to an overestimation of large root‐feeding nematodes, such as Hoplolaimidae species. The lack of quantification of nematodes inside the roots might form an additional bias in our data set, as numbers of endoparasitic nematodes may be underestimated when only soil samples are examined. Our combined methodological approach might allow future large‐scale nematode community analyses that include quantitative and qualitative information to be applied to compare nematode communities in any system. Furthermore, future improvements in sequencing technology will also enhance the taxonomic resolution to allow species or even strain‐level classification, such as pathotypes for root‐feeding nematodes, which will help identifying additional ecological patterns missed in this study.

## 5 CONCLUSION

Our results are among the first to test predictions on belowground community shifts due to intracontinental range expansions (Berg et al., 2010) and show that nematode community composition along a latitudinal transect of climate warming‐induced plant range expansion varies more strongly with latitude and plant species identity than with soil characteristics. The strength of nematode community shifts between the original and the new range of four range‐expanding plant species depended on plant species identity. Range expanders in general did not show stronger latitudinal community shifts than congeneric natives. We show that enemy release from root‐feeding nematodes may have occurred in one of the four range‐expanding plant species. Future studies should point out whether such potential enemy release patterns during intracontinental range expansions can be equally strong as in intercontinental invasions.

## References

[gcb14657-bib-0001] Bahram, M. , Hildebrand, F. , Forslund, S. K. , Anderson, J. L. , Soudzilovskaia, N. A. , Bodegom, P. M. , … Bork, P. (2018). Structure and function of the global topsoil microbiome. Nature, 560(7717), 233–237. 10.1038/s41586-018-0386-6 30069051

[gcb14657-bib-0002] Bates, S. T. , Clemente, J. C. , Flores, G. E. , Walters, W. A. , Parfrey, L. W. , Knight, R. , & Fierer, N. (2013). Global biogeography of highly diverse protistan communities in soil. ISME Journal, 7(3), 652–659. 23235291 10.1038/ismej.2012.147 PMC3578557

[gcb14657-bib-0003] Berg, M. P. , Kiers, E. T. , Driessen, G. , van der Heijden, M. , Kooi, B. W. , Kuenen, F. , … Ellers, J. (2010). Adapt or disperse: Understanding species persistence in a changing world. Global Change Biology, 16(2), 587–598. 10.1111/j.1365-2486.2009.02014.x

[gcb14657-bib-0004] Bezemer, T. , Fountain, M. , Barea, J. , Christensen, S. , Dekker, S. , Duyts, H. , … Maraun, M. (2010). Divergent composition but similar function of soil food webs of individual plants: Plant species and community effects. Ecology, 91(10), 3027–3036. 21058562 10.1890/09-2198.1

[gcb14657-bib-0005] Bhusal, D. R. , Tsiafouli, M. A. , & Sgardelis, S. P. (2015). Temperature‐based bioclimatic parameters can predict nematode metabolic footprints. Oecologia, 179(1), 187–199. 10.1007/s00442-015-3316-4 25899615

[gcb14657-bib-0006] Bongers, T. (1988). De nematoden van Nederland: een identificatietabel voor de in Nederland aangetroffen zoetwater‐en bodembewonende nematoden. Vereniging K. N. N. (Ed.). Zeist, The Netherlands: Koninklijke Nederlandse Natuurhistorische Vereniging.

[gcb14657-bib-0007] Callaway, R. M. , Cipollini, D. , Barto, K. , Thelen, G. C. , Hallett, S. G. , Prati, D. , … Klironomos, J. (2008). Novel weapons: Invasive plant suppresses fungal mutualists in America but not in its native Europe. Ecology, 89(4), 1043–1055. 10.1890/07-0370.1 18481529

[gcb14657-bib-0008] Chen, D. , Cheng, J. , Chu, P. , Hu, S. , Xie, Y. , Tuvshintogtokh, I. , & Bai, Y. (2015). Regional‐scale patterns of soil microbes and nematodes across grasslands on the Mongolian plateau: Relationships with climate, soil, and plants. Ecography, 38(6), 622–631. 10.1111/ecog.01226

[gcb14657-bib-0009] Darby, B. , Todd, T. C. , & Herman, M. A. (2013). High‐throughput amplicon sequencing of rRNA genes requires a copy number correction to accurately reflect the effects of management practices on soil nematode community structure. Molecular Ecology, 22(21), 5456–5471. 24103081 10.1111/mec.12480

[gcb14657-bib-0010] Dassen, S. , Cortois, R. , Martens, H. , de Hollander, M. , Kowalchuk, G. A. , van der Putten, W. H. , & De Deyn, G. B. (2017). Differential responses of soil bacteria, fungi, archaea and protists to plant species richness and plant functional group identity. Molecular Ecology, 26(15), 4085–4098. 10.1111/mec.14175 28489329

[gcb14657-bib-0011] De Deyn, G. B. , Raaijmakers, C. E. , Zoomer, H. R. , Berg, M. P. , de Ruiter, P. C. , Verhoef, H. A. , … van der Putten, W. H. (2003). Soil invertebrate fauna enhances grassland succession and diversity. Nature, 422(6933), 711–713. 10.1038/nature01548 12700759

[gcb14657-bib-0012] De Frenne, P. , Coomes, D. A. , De Schrijver, A. , Staelens, J. , Alexander, J. M. , Bernhardt‐Römermann, M. , … Verheyen, K. (2014). Plant movements and climate warming: Intraspecific variation in growth responses to nonlocal soils. New Phytologist, 202(2), 431–441. 10.1111/nph.12672 24387238

[gcb14657-bib-0013] de Hollander, M. (2017). nioo‐knaw/hydra: 1.3.3 (Version 1.3.3). Retrieved from https://zenodo.org/record/884028#.XKw9spgzaUk: Zenodo

[gcb14657-bib-0014] De Rosario‐Martinez, H. (2013). phia: Post‐hoc interaction analysis. R package version 0.1‐3.

[gcb14657-bib-0015] de Ruiter, P. C. , Neutel, A.‐M. , & Moore, J. C. (1995). Energetics, patterns of interaction strengths, and stability in real ecosystems. Science, 269(5228), 1257–1260. 10.1126/science.269.5228.1257 17732112

[gcb14657-bib-0016] Delgado‐Baquerizo, M. , Oliverio, A. M. , Brewer, T. E. , Benavent‐González, A. , Eldridge, D. J. , Bardgett, R. D. , … Fierer, N. (2018). A global atlas of the dominant bacteria found in soil. Science, 359(6373), 320–325. 29348236 10.1126/science.aap9516

[gcb14657-bib-0017] Dostálek, T. , Münzbergová, Z. , Kladivová, A. , & Macel, M. (2015). Plant–soil feedback in native vs. invasive populations of a range expanding plant. Plant and Soil, 399(1), 1–12.

[gcb14657-bib-0018] Edgar, R. C. (2010). Search and clustering orders of magnitude faster than BLAST. Bioinformatics, 26(19), 2460–2461. 10.1093/bioinformatics/btq461 20709691

[gcb14657-bib-0019] Edgar, R. C. , Haas, B. J. , Clemente, J. C. , Quince, C. , & Knight, R. (2011). UCHIME improves sensitivity and speed of chimera detection. Bioinformatics, 27(16), 2194–2200. 10.1093/bioinformatics/btr381 21700674 PMC3150044

[gcb14657-bib-0020] Engelkes, T. , Morrien, E. , Verhoeven, K. J. F. , Bezemer, T. M. , Biere, A. , Harvey, J. A. , … van der Putten, W. H. (2008). Successful range‐expanding plants experience less above‐ground and below‐ground enemy impact. Nature, 456(7224), 946–948. 10.1038/nature07474 19020504

[gcb14657-bib-0021] Ettema, C. H. , Rathbun, S. L. , & Coleman, D. C. (2000). On spatiotemporal patchiness and the coexistence of five species of Chronogaster (Nematoda: Chronogasteridae) in a riparian wetland. Oecologia, 125(3), 444–452. 28547340 10.1007/s004420000468

[gcb14657-bib-0022] Ferris, H. , Bongers, T. , & De Goede, R. (2001). A framework for soil food web diagnostics: Extension of the nematode faunal analysis concept. Applied Soil Ecology, 18(1), 13–29.

[gcb14657-bib-0023] Fierer, N. , Strickland, M. S. , Liptzin, D. , Bradford, M. A. , & Cleveland, C. C. (2009). Global patterns in belowground communities. Ecology Letters, 12(11), 1238–1249. 19674041 10.1111/j.1461-0248.2009.01360.x

[gcb14657-bib-0024] Foissner, W. , & Hawksworth, D. L. (2009). Protist diversity and geographical distribution (Vol. 8). Berlin, Germany: Springer Science & Business Media.

[gcb14657-bib-0025] Geisen, S. , Kostenko, O. , Cnossen, M. C. , ten Hooven, F. C. , Vreš, B. , & van der Putten, W. H. (2017). Seed and root endophytic fungi in a range expanding and a related plant species. Frontiers in Microbiology, 8, 1645 10.3389/fmicb.2017.01645 28900420 PMC5581836

[gcb14657-bib-0026] Geisen, S. , Snoek, L. B. , ten Hooven, F. C. , Duyts, H. , Kostenko, O. , Bloem, J. , … van der Putten, W. H. (2018). Integrating quantitative morphological and qualitative molecular methods to analyse soil nematode community responses to plant range expansion. Methods in Ecology and Evolution, 9(6), 1366–1378. 10.1111/2041-210x.12999

[gcb14657-bib-0027] Griffiths, B. S. , de Groot, G. A. , Laros, I. , Stone, D. , & Geisen, S. (2018). The need for standardisation: Exemplified by a description of the diversity, community structure and ecological indices of soil nematodes. Ecological Indicators, 87, 43–46. 10.1016/j.ecolind.2017.12.002

[gcb14657-bib-0028] Guillou, L. , Bachar, D. , Audic, S. , Bass, D. , Berney, C. , Bittner, L. , … Christen, R. (2013). The Protist Ribosomal Reference database (PR2): A catalog of unicellular eukaryote small sub‐unit rRNA sequences with curated taxonomy. Nucleic Acids Research, 41(D1), D597–D604. 10.1093/nar/gks1160 23193267 PMC3531120

[gcb14657-bib-0029] Hu, C. , & Qi, Y. (2010). Effect of compost and chemical fertilizer on soil nematode community in a Chinese maize field. European Journal of Soil Biology, 46(3), 230–236. 10.1016/j.ejsobi.2010.04.002

[gcb14657-bib-0030] Kardol, P. , Bezemer, T. M. , & van der Putten, W. H. (2006). Temporal variation in plant–soil feedback controls succession. Ecology Letters, 9(9), 1080–1088. 10.1111/j.1461-0248.2006.00953.x 16925657

[gcb14657-bib-0031] Kerfahi, D. , Tripathi, B. M. , Porazinska, D. L. , Park, J. , Go, R. , & Adams, J. M. (2016). Do tropical rain forest soils have greater nematode diversity than High Arctic tundra? A metagenetic comparison of Malaysia and Svalbard. Global Ecology and Biogeography, 25(6), 716–728.

[gcb14657-bib-0032] Köster, J. , & Rahmann, S. (2012). Snakemake—A scalable bioinformatics workflow engine. Bioinformatics, 28(19), 2520–2522. 10.1093/bioinformatics/bts480 22908215

[gcb14657-bib-0033] Lauber, C. L. , Hamady, M. , Knight, R. , & Fierer, N. (2009). Pyrosequencing‐based assessment of soil pH as a predictor of soil bacterial community structure at the continental scale. Applied and Environmental Microbiology, 75(15), 5111–5120. 19502440 10.1128/AEM.00335-09 PMC2725504

[gcb14657-bib-0034] Lu, X. , He, M. , Ding, J. , & Siemann, E. (2018). Latitudinal variation in soil biota: Testing the biotic interaction hypothesis with an invasive plant and a native congener. ISME Journal, 12, 2811–2822. 10.1038/s41396-018-0219-5 30013163 PMC6246596

[gcb14657-bib-0035] Martin, M. (2011). Cutadapt removes adapter sequences from high‐throughput sequencing reads. Embnet Journal, 17(1), 10–12.

[gcb14657-bib-0036] Morriën, E. , Duyts, H. , & van der Putten, W. H. (2012). Effects of native and exotic range‐expanding plant species on taxonomic and functional composition of nematodes in the soil food web. Oikos, 121(2), 181–190. 10.1111/j.1600-0706.2011.19773.x

[gcb14657-bib-0037] Morriën, E. , Engelkes, T. , Macel, M. , Meisner, A. , & van der Putten, W. H. (2010). Climate change and invasion by intracontinental range‐expanding exotic plants: The role of biotic interactions. Annals of Botany, 105(6), 843–848. 10.1093/aob/mcq064 20354072 PMC2876007

[gcb14657-bib-0038] Morriën, E. , & van der Putten, W. H. (2013). Soil microbial community structure of range‐expanding plant species differs from co‐occurring natives. Journal of Ecology, 101(5), 1093–1102. 10.1111/1365-2745.12117

[gcb14657-bib-0039] NDFF (2017). Verspreidingsatlas planten. Retrieved from http://www.verspreidingsatlas.nl/planten

[gcb14657-bib-0040] Nicol, J. M. , Turner, S. J. , Coyne, D. L. , Nijs, L. D. , Hockland, S. , & Maafi, Z. T. (2011). Current nematode threats to World agriculture In Jones J., Gheysen G., & Fenoll C. (Eds.), Genomics and molecular genetics of plant‐nematode interactions (pp. 21–43). Dordrecht, the Netherlands: Springer Netherlands.

[gcb14657-bib-0041] Nielsen, U. N. , Ayres, E. , Wall, D. H. , Li, G. , Bardgett, R. D. , Wu, T. , & Garey, J. R. (2014). Global‐scale patterns of assemblage structure of soil nematodes in relation to climate and ecosystem properties. Global Ecology and Biogeography, 23(9), 968–978. 10.1111/geb.12177

[gcb14657-bib-0042] Oostenbrink, M. (1960). Estimating nematode populations by some elected methods In Sasser J. N. &Jenkins W. R. (Eds.), Nematology (pp. 85–102). Chapel Hill, NC: Univ. of North Carolina Press.

[gcb14657-bib-0043] Parmesan, C. , & Yohe, G. (2003). A globally coherent fingerprint of climate change impacts across natural systems. Nature, 421(6918), 37–42. 10.1038/nature01286 12511946

[gcb14657-bib-0044] Pawlowski, J. , Audic, S. , Adl, S. , Bass, D. , Belbahri, L. , Berney, C. , … de Vargas, C. (2012). CBOL protist working group: Barcoding eukaryotic richness beyond the animal, plant, and fungal kingdoms. PLOS Biology, 10(11), e1001419 10.1371/journal.pbio.1001419 23139639 PMC3491025

[gcb14657-bib-0045] Quist, C. W. (2017). Impact of trophic ecologies on the whereabouts of nematodes in soil. Wageningen, the Netherlands: Wageningen University Retrieved from http://edepot.wur.nl/403954

[gcb14657-bib-0046] Quist, C. W. , Gort, G. , Mulder, C. , Wilbers, R. H. P. , Termorshuizen, A. J. , Bakker, J. , & Helder, J. (2017). Feeding preference as a main determinant of microscale patchiness among terrestrial nematodes. Molecular Ecology Resources, 17(6), 1257–1270. 10.1111/1755-0998.12672 28323394

[gcb14657-bib-0047] R Core Development Team . (2012). R: A language and environment for statistical computing. Vienna, Austria: R Foundation for Statistical Computing.

[gcb14657-bib-0048] Ripley, B. , Venables, B. , Bates, D. M. , Hornik, K. , Gebhardt, A. , Firth, D. , & Ripley, M. B. (2013). Package ‘mass’. Cran R.

[gcb14657-bib-0049] Rognes, T. , Flouri, T. , Nichols, B. , Quince, C. , & Mahé, F. (2016). VSEARCH: A versatile open source tool for metagenomics. PeerJ Preprints, 4, e2409v2401 10.7287/peerj.preprints.2409v1 27781170 PMC5075697

[gcb14657-bib-0050] Rumpf, S. B. , Hülber, K. , Klonner, G. , Moser, D. , Schütz, M. , Wessely, J. , … Dullinger, S. (2018). Range dynamics of mountain plants decrease with elevation. Proceedings of the National Academy of Sciences USA, 115(8), 1848–1853. 10.1073/pnas.1713936115 PMC5828587 29378939

[gcb14657-bib-0051] Schaffner, U. , Ridenour, W. M. , Wolf, V. C. , Bassett, T. , Mueller, C. , Mueller‐Schaerer, H. , … Callaway, R. M. (2011). Plant invasions, generalist herbivores, and novel defense weapons. Ecology, 92(4), 829–835. 10.1890/10-1230.1 21661546

[gcb14657-bib-0052] Šmilauer, P. , & Lepš, J. (2014). Multivariate analysis of ecological data using CANOCO 5. Cambridge, UK: Cambridge University Press.

[gcb14657-bib-0053] Song, D. , Pan, K. , Tariq, A. , Sun, F. , Li, Z. , Sun, X. , … Wu, X. (2017). Large‐scale patterns of distribution and diversity of terrestrial nematodes. Applied Soil Ecology, 114, 161–169.

[gcb14657-bib-0054] Steinbauer, M. J. , Grytnes, J.‐A. , Jurasinski, G. , Kulonen, A. , Lenoir, J. , Pauli, H. , … Wipf, S. (2018). Accelerated increase in plant species richness on mountain summits is linked to warming. Nature, 556, 231–234. 10.1038/s41586-018-0005-6 29618821

[gcb14657-bib-0055] Sylvain, Z. A. , Wall, D. H. , Cherwin, K. L. , Peters, D. P. C. , Reichmann, L. G. , & Sala, O. E. (2014). Soil animal responses to moisture availability are largely scale, not ecosystem dependent: Insight from a cross‐site study. Global Change Biology, 20(8), 2631–2643. 10.1111/gcb.12522 24399762

[gcb14657-bib-0056] Tedersoo, L. , Bahram, M. , Põlme, S. , Kõljalg, U. , Yorou, N. S. , Wijesundera, R. , … Suija, A. (2014). Global diversity and geography of soil fungi. Science, 346(6213), 1256688. 25430773 10.1126/science.1256688

[gcb14657-bib-0057] Ter Braak, C. , & Šmilauer, P. (2012). Canoco 5, Windows release (5.00). Software for multivariate data exploration, testing, and summarization. Biometris, Plant Research International, Wageningen, the Netherlands.

[gcb14657-bib-0058] Thompson, L. R. , Sanders, J. G. , McDonald, D. , Amir, A. , Ladau, J. , Locey, K. J. , … Ackermann, G. (2017). A communal catalogue reveals Earth's multiscale microbial diversity. Nature, 551(7681), 457–463. 29088705 10.1038/nature24621 PMC6192678

[gcb14657-bib-0059] van der Heijden, M. G. A. , Bardgett, R. D. , & van Straalen, N. M. (2008). The unseen majority: Soil microbes as drivers of plant diversity and productivity in terrestrial ecosystems. Ecology Letters, 11(3), 296–310. 10.1111/j.1461-0248.2007.01139.x 18047587

[gcb14657-bib-0060] Van der Meijden, R. (2005). Heukels Flora van Nederland 23e druk. Groningen, The Netherlands: Wolters‐Noordhoff.

[gcb14657-bib-0061] van der Putten, W. H. (2012). Climate change, aboveground‐belowground interactions, and species' range shifts. Annual Review of Ecology, Evolution, and Systematics, 43, 365–383. 10.1146/annurev-ecolsys-110411-160423

[gcb14657-bib-0062] van der Putten, W. H. , Yeates, G. W. , Duyts, H. , Reis, C. S. , & Karssen, G. (2005). Invasive plants and their escape from root herbivory: A worldwide comparison of the root‐feeding nematode communities of the dune grass *Ammophila arenaria* in natural and introduced ranges. Biological Invasions, 7(4), 733–746. 10.1007/s10530-004-1196-3

[gcb14657-bib-0063] van Grunsven, R. H. A. , van der Putten, W. H. , Bezemer, T. M. , Berendse, F. , & Veenendaal, E. M. (2010). Plant‐soil interactions in the expansion and native range of a poleward shifting plant species. Global Change Biology, 16(1), 380–385. 10.1111/j.1365-2486.2009.01996.x

[gcb14657-bib-0064] Van Nuland, M. E. , Bailey, J. K. , & Schweitzer, J. A. (2017). Divergent plant–soil feedbacks could alter future elevation ranges and ecosystem dynamics. Nature Ecology & Evolution, 1, 0150. 10.1038/s41559-017-0150 28812635

[gcb14657-bib-0065] Vandeputte, D. , Kathagen, G. , D'hoe, K. , Vieira‐Silva, S. , Valles‐Colomer, M. , Sabino, J. , … Raes, J. (2017). Quantitative microbiome profiling links gut community variation to microbial load. Nature, 551, 507–511. 10.1038/nature24460 29143816

[gcb14657-bib-0066] Wilkinson, D. M. , Koumoutsaris, S. , Mitchell, E. A. D. , & Bey, I. (2012). Modelling the effect of size on the aerial dispersal of microorganisms. Journal of Biogeography, 39(1), 89–97. 10.1111/j.1365-2699.2011.02569.x

[gcb14657-bib-0067] Wilschut, R. A. , Kostenko, O. , Koorem, K. , & van der Putten, W. H. (2018). Nematode community responses to range‐expanding and native plant communities in original and new range soils. Ecology and Evolution, 8(20), 10288–10297. 10.1002/ece3.4505 30397466 PMC6206179

[gcb14657-bib-0068] Wilschut, R. A. , Silva, J. C. P. , Garbeva, P. , & van der Putten, W. H. (2017). Belowground plant‐herbivore interactions vary among climate‐driven range‐expanding plant species with different degrees of novel chemistry. Frontiers Plant Science, 8, 1861 10.3389/fpls.2017.01861 PMC5660973 29118781

[gcb14657-bib-0069] Yeates, G. W. , Bongers, T. , Degoede, R. G. M. , Freckman, D. W. , & Georgieva, S. S. (1993). Feeding‐habits in soil nematode families and genera – An outline for soil ecologists. Journal of Nematology, 25(3), 315–331. 10.1890/15-1285.1 19279775 PMC2619405

[gcb14657-bib-0070] Zhao, J. , Wang, F. , Li, J. , Zou, B. , Wang, X. , Li, Z. , & Fu, S. (2014). Effects of experimental nitrogen and/or phosphorus additions on soil nematode communities in a secondary tropical forest. Soil Biology and Biochemistry, 75, 1–10. 10.1016/j.soilbio.2014.03.019

[gcb14657-bib-0071] Zhu, F. , Massana, R. , Not, F. , Marie, D. , & Vaulot, D. (2005). Mapping of picoeucaryotes in marine ecosystems with quantitative PCR of the 18S rRNA gene. FEMS Microbiology Ecology, 52(1), 79–92. 10.1016/j.femsec.2004.10.006 16329895

