# Supplementary material for: Latitudinal variation in soil nematode communities under climate warming‐related range‐expanding and native plants
Source: Glob Chang Biol. 2019 May 20;25(8):2714–26. doi: 10.1111/gcb.14657 (PMC6617783; doi:10.1111/gcb.14657)
Supplement: Supplementary file 1 [file GCB-25-2714-s001.docx]

**Supplementary information**

**Latitudinal variation in soil nematode communities under climate warming-related range-expanding and native plants**

*Rutger A. Wilschut^1,2*^, Stefan Geisen^1^, Henk Martens^1^, Olga Kostenko^1^, Mattias de Hollander^1^, Freddy ten Hooven^1^, Carolin Weser^1^, L. Basten Snoek^1,3^, Janneke Bloem^1^, Danka Caković^4^, Tatjana Čelik^5^, Kadri Koorem^1,6^, Nikos Krigas^7,8^, Marta Manrubia^1^, Kelly S. Ramirez^1^, Maria A. Tsiafouli^7^, Branko Vreš^5^, & Wim H. van der Putten^1,2^*

# The Netherlands

**Latitude**


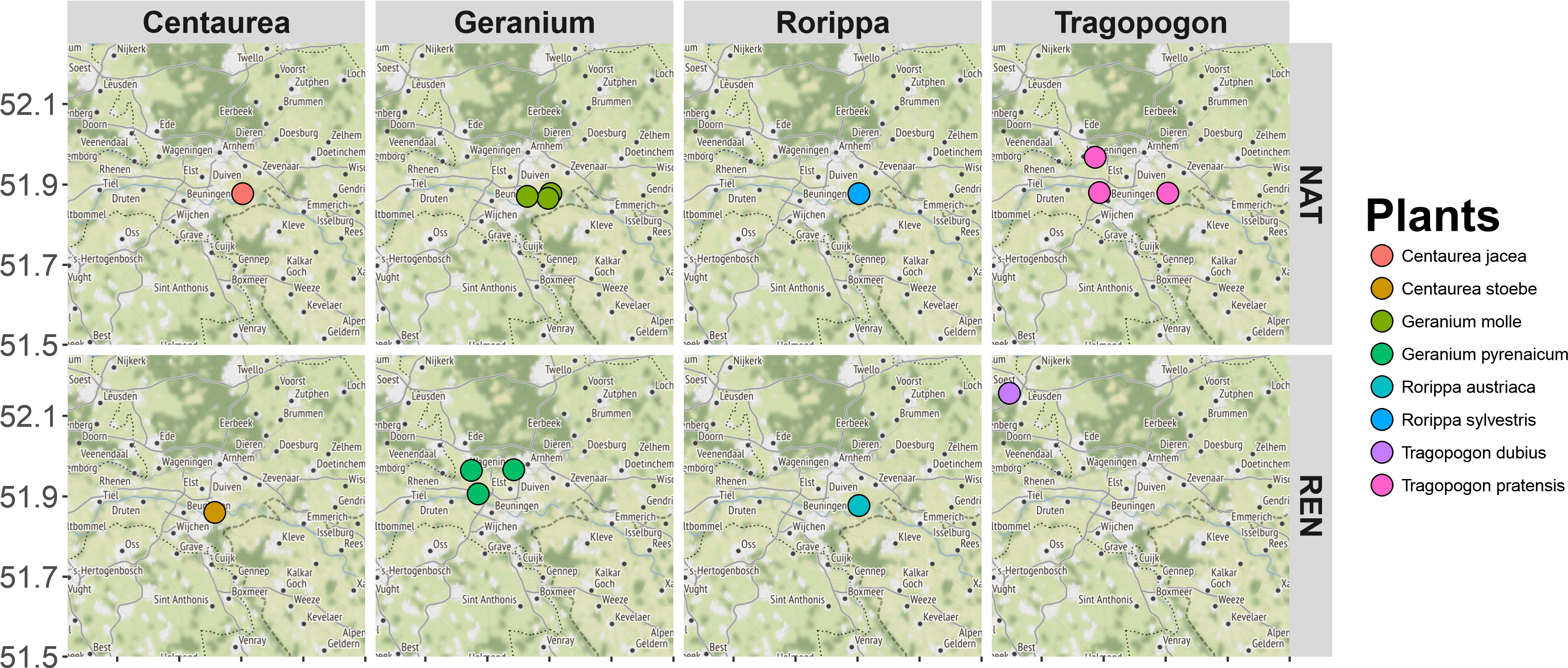


5.505.756.006.256.505.505.756.006.256.505.505.756.006.256.505.505.756.006.256.50

**Longitude**

#
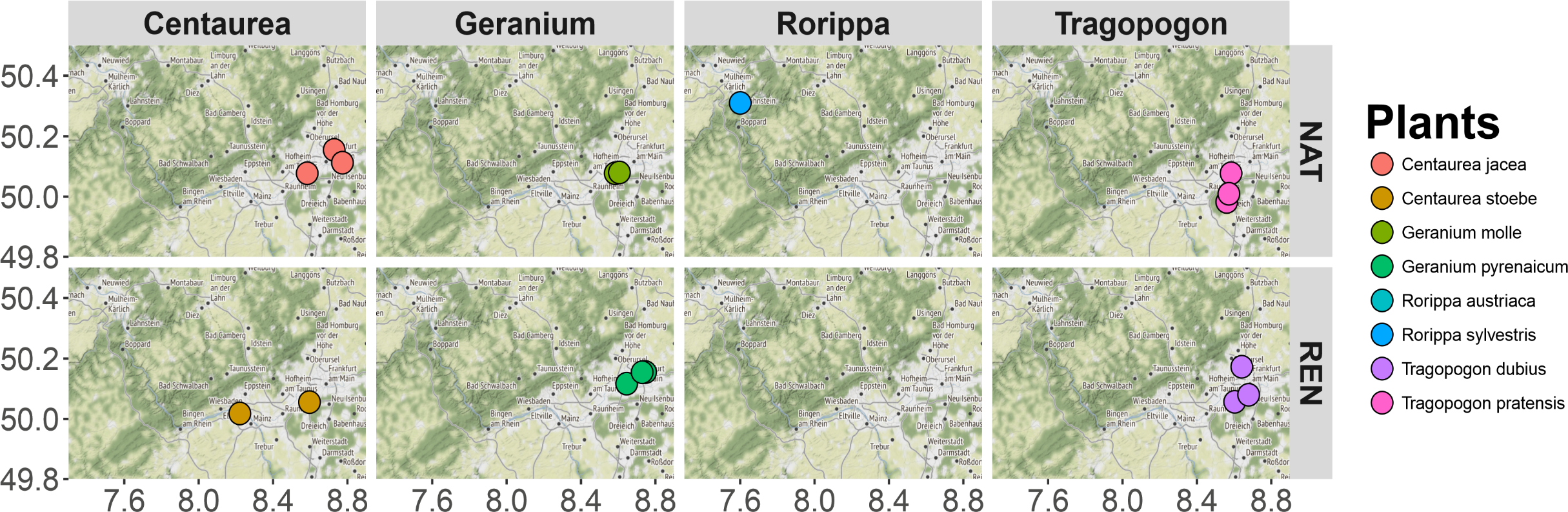
Germany

**Latitude**

**Longitude**

# Austria

**Latitude**


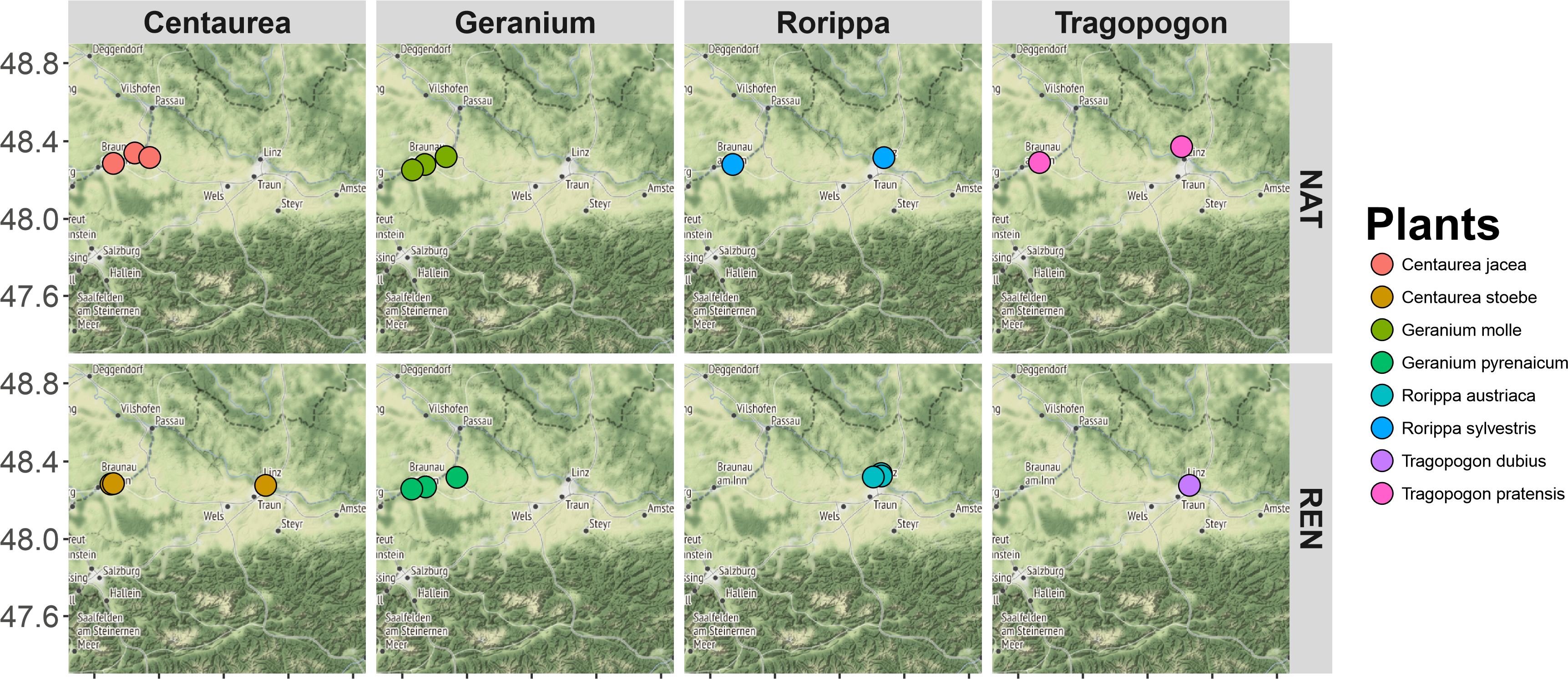


13.013.514.014.515.013.013.514.014.515.013.013.514.014.515.013.013.514.014.515.0 **Longitude**

# Slovenia


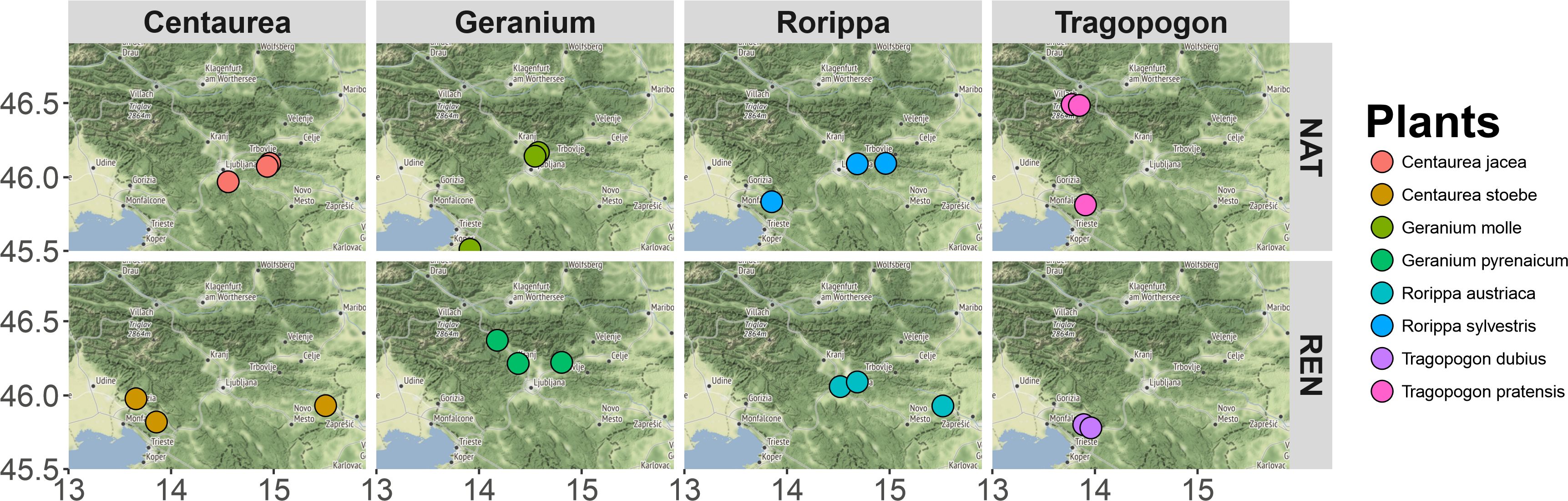


**Latitude**

**Longitude**

# Montenegro

**Latitude**


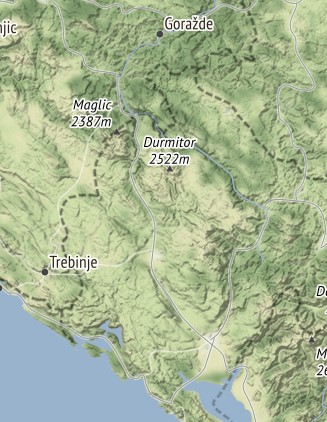


●

●

●


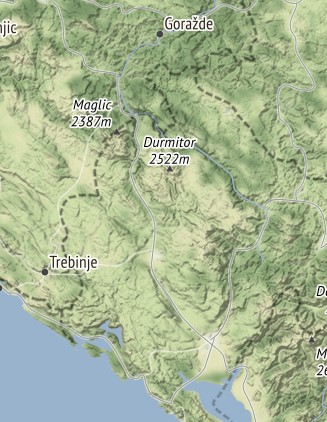

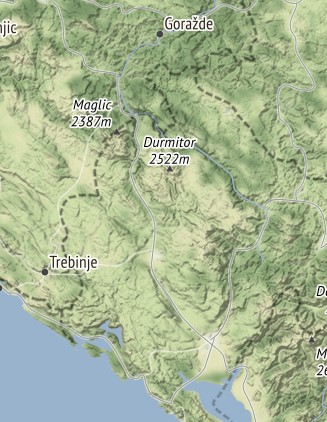


●

●


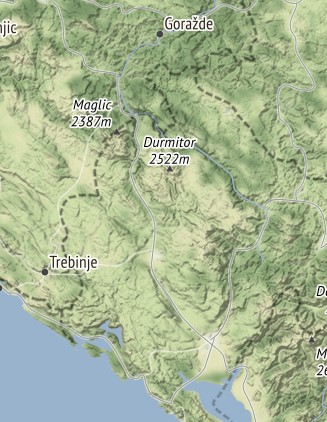


●

●

●


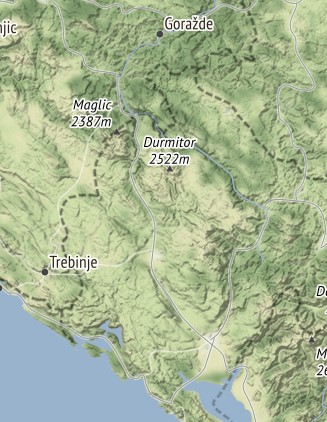


●

●

●

●


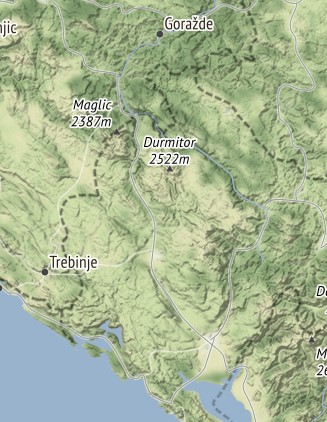

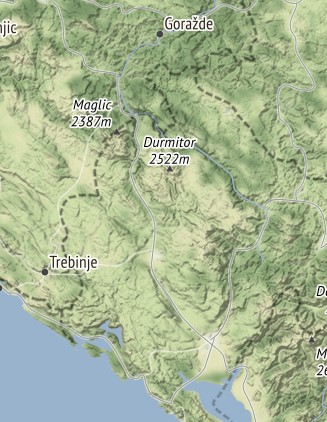


●

●


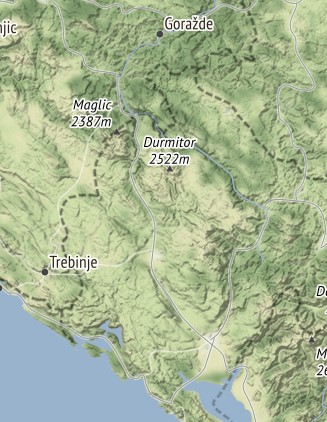


●

**Centaurea**

**Geranium**

**Rorippa**

**Tragopogon**

**NAT**

**REN**

18.5

19.0

19.5

18.5

19.0

19.5

18.5

19.0

19.5

18.5

19.0

19.5

42.5

43.0

43.5

42.5

43.0

43.5

**Plants**

●

●

●

●

●

●

●

●

Centaurea jacea

Centaurea stoebe

Geranium molle

Geranium pyrenaicum

Rorippa austriaca

Rorippa sylvestris

Tragopogon dubius

Tragopogon pratensis

**Longitude**

# Greece

**Latitude**


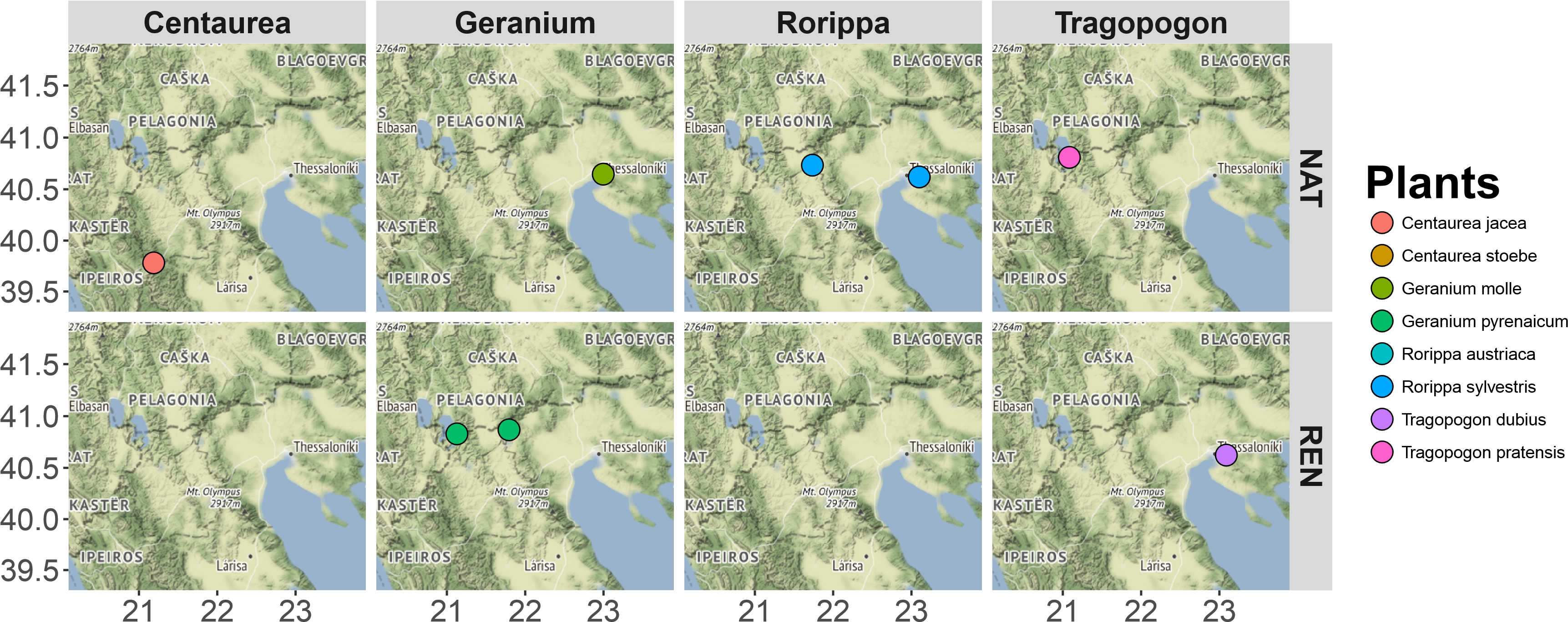


**Longitude**

**Supplementary Figure 1**: Sampling scheme of four native and four range-expanding plant species in European countries. For each plant species, coloured circles represent populations within each of the six countries where plants were sampled: Greece, Montenegro, Slovenia, Austria, Germany and The Netherlands. Note that range-expanding Centaurea and Rorippa were not present in Greece and Montenegro, and that dots may overlap due to relative proximity of the populations.


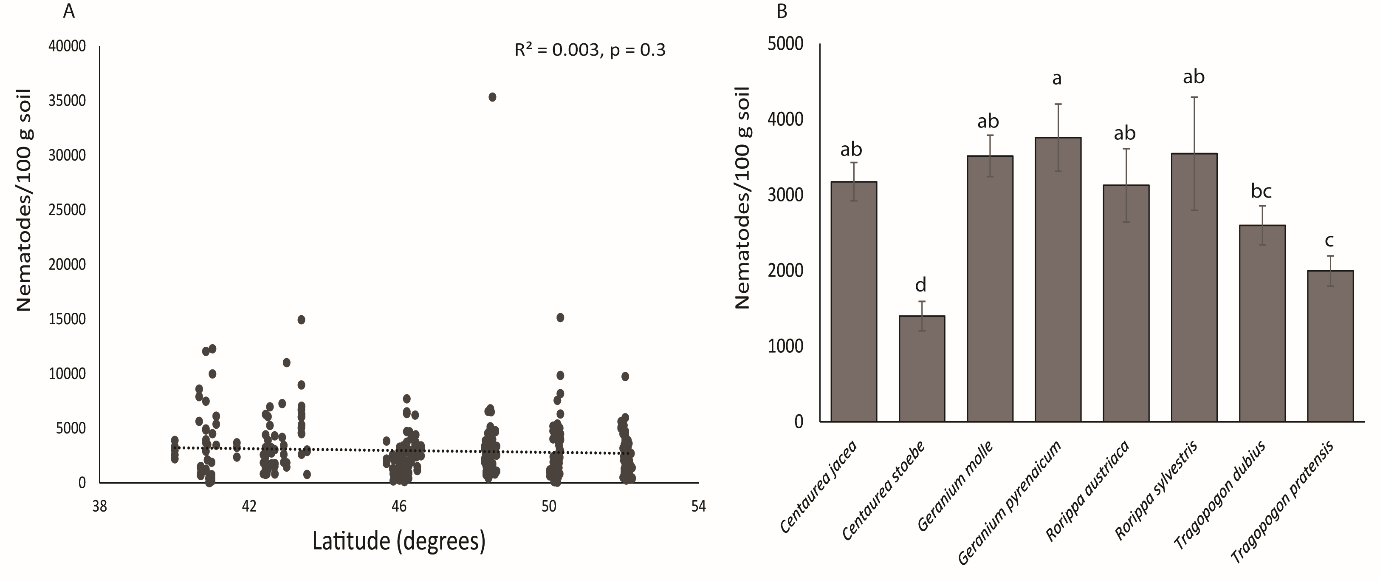


**Supplementary Figure 2**: A) Overall correlation between latitude and nematode abundance (N/100 g soil) along a latitudinal transect from Greece to The Netherlands. R^2^ and p-value of Pearson correlation test are shown. B) Nematode abundance in rhizosphere soil from native plant species *Centaurea jacea*, *Geranium molle, Rorrippa sylvestris*, and *Tragopogon pratensis* and congeneric range-expanding plant species *C. stoebe*, *G. pyrenaicum*, *R. austriaca* and *T. dubius*. Bars represent average (± s.e.) nematode numbers per 100 g dry weight soil and letters indicate statistical differences of Negative binomial GLM and post hoc Wald tests.


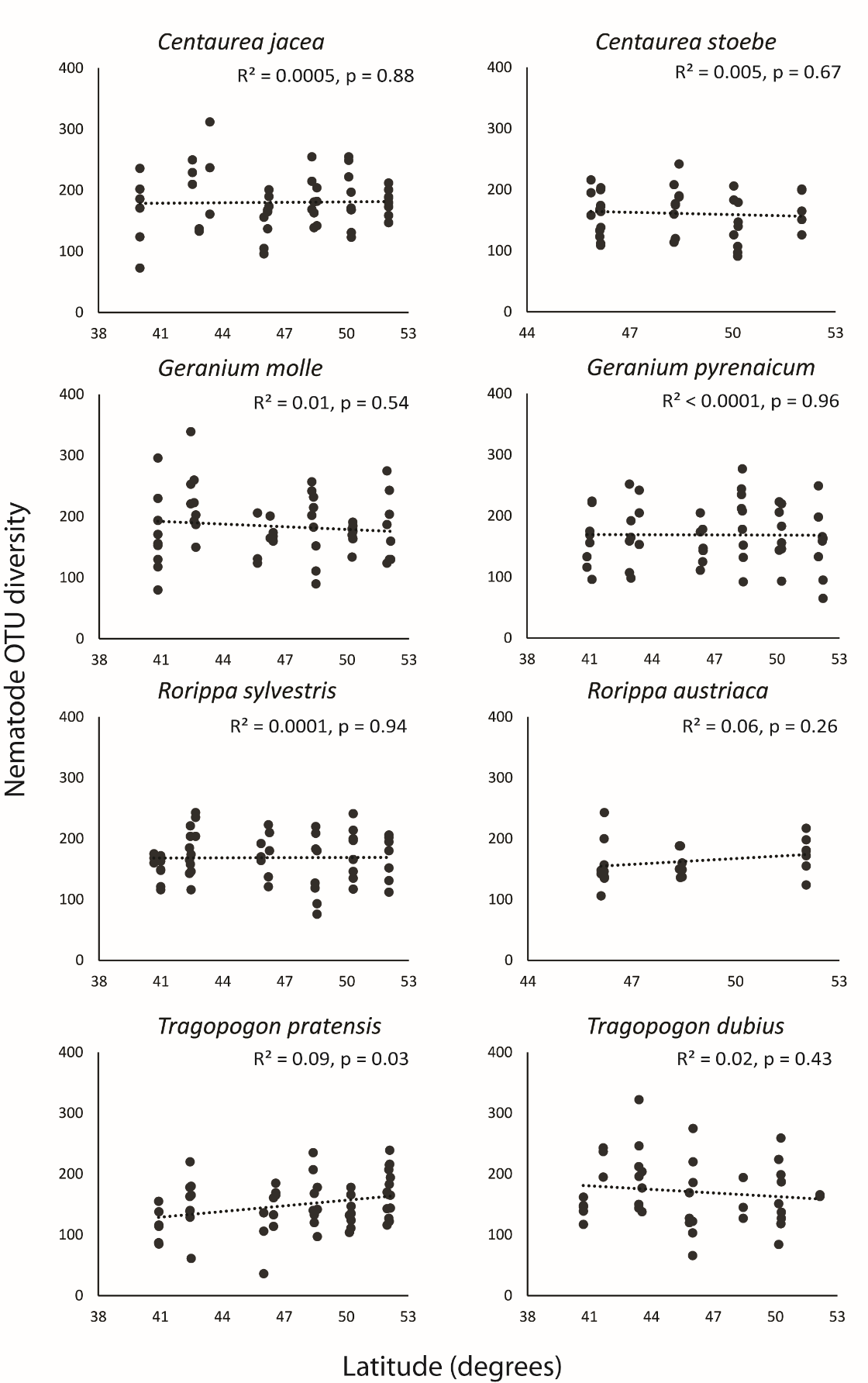


**Supplementary Figure 3**: Correlations between latitude and nematode OTU diversity in rhizosphere samples of four native plant species (left panels) and four congeneric range-expanding plant species (right panels). R^2^ and p-values of Pearson correlation tests are shown.


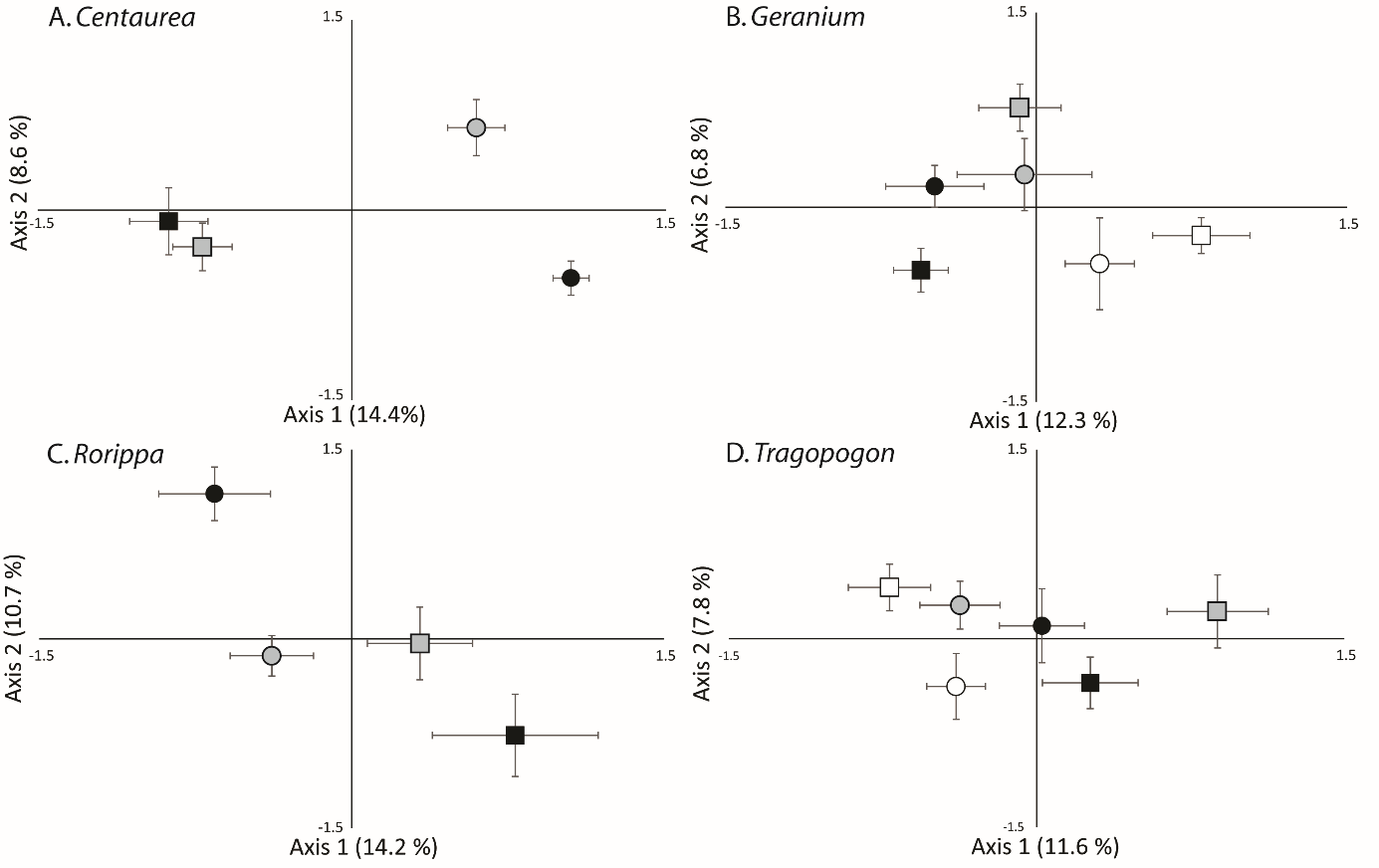


**Supplementary Figure 4** Ordination plots based on Principal component analyses (PCA) of nematode communities on genus-level in the rhizospheres of range-expanding (circular centroids) and native (rectangular centroids) *Centaurea* (A), *Geranium* (B), *Rorippa* (C), and *Tragopogon* (D). Sign colours represent southern latitude soils (white; Greece and Montengro), central latitude soils (grey; Slovenia and Austria) and northern latitude soils (black; Central-West Germany and The Netherlands).

**
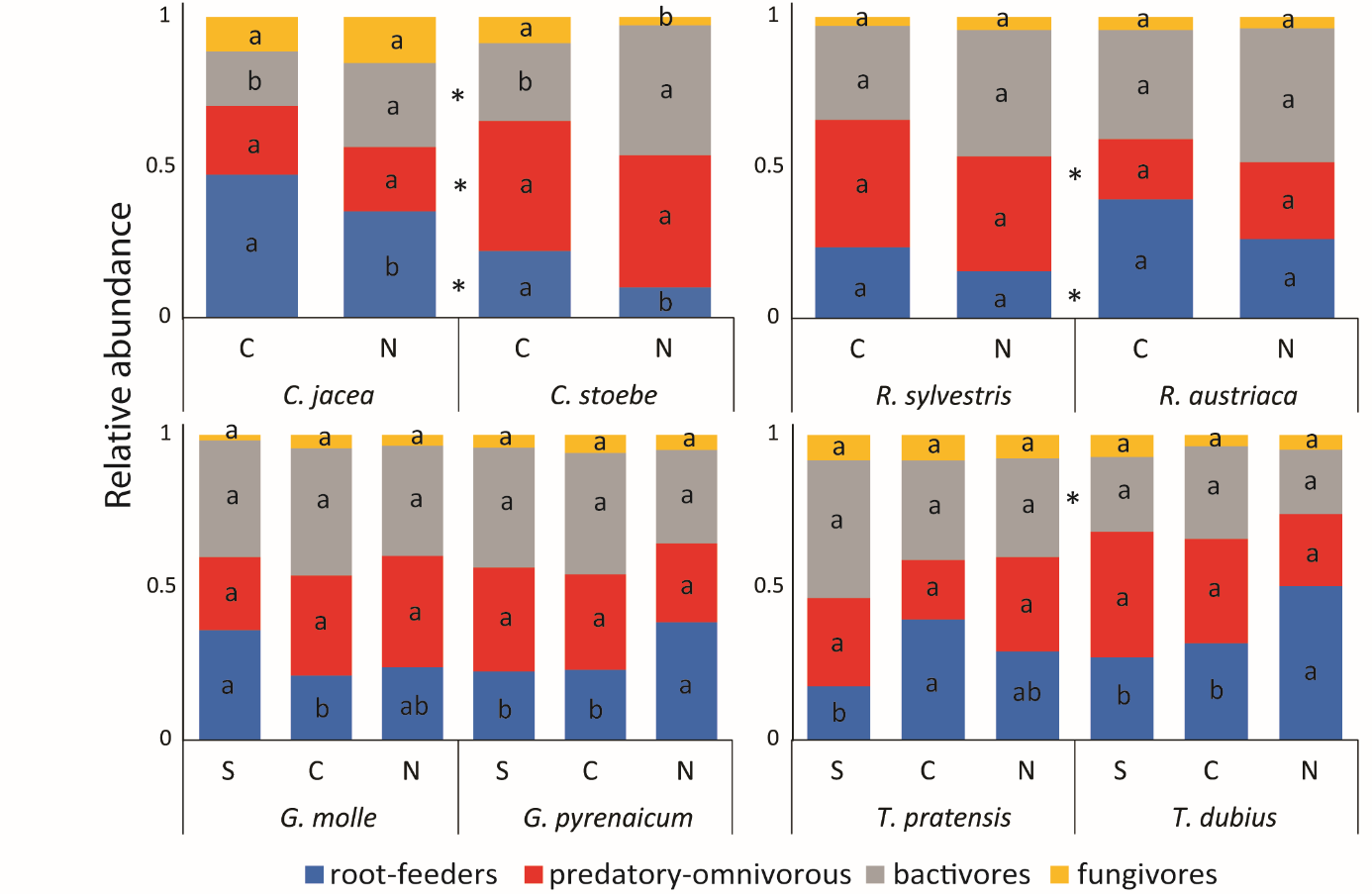
**

**Supplementary Figure 5** Relative abundances of four major nematode feeding groups in rhizosphere samples of range-expanding *Centaurea stoebe*, *Geranium pyrenaicum*, *Rorippa austriaca* and *Tragopogon dubius*, and of the native plant species *Centaurea jacea*, *Geranium molle*, *Rorippa sylvestris* and *Tragopogon pratensis* in southern (S: Greece and Montengro; only *Geranium* and *Tragopogon*) and central (C: Slovenia and Austria) original range soils and new range soils (N: Central-West Germany and The Netherlands). Small letters indicate significant within-species differences according to post-hoc Wald tests. Significant between-species differences of nematode feeding type abundances are indicated with *.

**Supplementary Table 1**: Results of the Redundancy Analyses (RDA) with forward selection on the composition of nematode communities based on nematode OTUs or nematode genera. Factors contributing more than 5% to the explained variation by the RDA-model have been listed.

|  |  |  | |  |  |  |
| --- | --- | --- | --- | --- | --- | --- |
| Database: OTU-level | Explains % | | Contribution % | Pseudo-F | P | P(adj) |
| Latitude | 2.1 | | 15.6 | 6.0 | 0.002 | 0.03 |
| Phosphate | 1.3 | | 9.5 | 3.7 | 0.002 | 0.03 |
| Plant species: *Centaurea stoebe* | 1.2 | | 9.0 | 3.5 | 0.002 | 0.03 |
| Soil moisture | 1.1 | | 8.5 | 3.4 | 0.002 | 0.03 |
| pH | 1.1 | | 8.4 | 3.3 | 0.002 | 0.03 |
| Plant species: *Rorippa sylvestris* | 1.1 | | 8.0 | 3.2 | 0.002 | 0.03 |
| Plant species: *Tragopogon pratensis* | 0.9 | | 6.9 | 2.8 | 0.002 | 0.03 |
| Plant species: *Centaurea jacea* | 0.8 | | 6.0 | 2.4 | 0.002 | 0.03 |
| Plant species: *Geranium pyrenaicum* | 0.8 | | 6.0 | 2.4 | 0.002 | 0.03 |
| $\mathrm{NO}_{2}^{-}$+ $\mathrm{NO}_{3}^{-}$ | 0.7 | | 5.5 | 2.2 | 0.002 | 0.03 |
| Plant species: *Rorippa austriaca* | 0.7 | | 5.2 | 2.1 | 0.002 | 0.03 |

| Database: Genus-level | Explains % | Contribution % | Pseudo-F | P | P(adj) |
| --- | --- | --- | --- | --- | --- |
| Latitude | 3.1 | 19.1 | 8.9 | 0.002 | 0.03 |
| Phosphate | 1.6 | 10.0 | 4.7 | 0.002 | 0.03 |
| Soil moisture | 1.8 | 11.0 | 5.3 | 0.002 | 0.03 |
| Plant species: *Centaurea stoebe* | 1.5 | 9.0 | 4.4 | 0.002 | 0.03 |
| pH | 1.3 | 8.0 | 3.9 | 0.002 | 0.03 |
| Plant species: *Rorippa sylvestris* | 1.2 | 7.3 | 3.6 | 0.002 | 0.03 |
| $\mathrm{NO}_{2}^{-}$+ $\mathrm{NO}_{3}^{-}$ | 1.1 | 6.6 | 3.3 | 0.002 | 0.03 |
| Plant species: *Tragopogon pratensis* | 0.8 | 5.1 | 2.5 | 0.002 | 0.03 |
